# Electron Density and Molecular Orbital Analyses of the Nature of Bonding in the η^3^-CCH Agostic Rhodium Complexes Preceding the C–C and C–H Bond Cleavages

**DOI:** 10.3390/molecules29204788

**Published:** 2024-10-10

**Authors:** Irena Efremenko

**Affiliations:** Department of Molecular Chemistry and Materials Science, Weizmann Institute of Science, Rehovot 7610001, Israel; irena.efremenko@weizmann.ac.il

**Keywords:** Rh pincer complexes, agostic bonding, η^3^-CCH agostic intermediates, C–C and C–H bond activation, electron density analysis, QTAIM analysis, molecular orbital analysis, dynamic bonding behavior

## Abstract

In our recent work, we revisited C–H and C–C bond activation in rhodium (I) complexes of pincer ligands PCP, PCN, PCO, POCOP, and SCS. Our findings indicated that an η^3^-C_sp2_C_sp3_H agostic intermediate acts as a common precursor to both C–C and C–H bond activation in these systems. We explore the electronic structure and bonding nature of these precleavage complexes using electron density and molecular orbital analyses. Using NBO, IBO, and ESI-3D methods, the bonding in the η^3^-CCH agostic moiety is depicted by two three-center agostic bonds: Rh–C_sp2_–C_sp3_ and Rh–C_sp3_–H, with all three atoms datively bound to Rh(I). IBO analysis specifically highlights the involvement of three orbitals (CC→Rh and CH→Rh σ donation, plus Rh→CCH π backdonation) in both C–C and C–H bond cleavages. NCIPLOT and QTAIM analyses highlight anagostic (Rh–H) or β-agostic (Rh–C_sp2_–H) interactions and the absence of Rh–C_sp_^3^ interactions. QTAIM molecular graphs suggest bond path instability under dynamic conditions due to the nearness of line and ring critical points. Several low-frequency and low-force vibrational modes interconvert various bonding patterns, reinforcing the dynamic η^3^-CCH agostic nature. The kinetic preference for C–H bond breaking is attributed to the smaller reduced mass of C–H vibrations compared to C–C vibrations.

## 1. Introduction

Understanding the mechanisms underlying the activation of strong C–H and C–C bonds by transition metals is a fundamental subject of interest in organometallic chemistry. The preliminary coordination of the substrate is a mandatory step in these reactions, often determining the kinetics and overall directions of the processes [1]. Agostic complexes are typical intermediates in C–H bond activation. Initially defined as complexes involving covalent interactions between a C–H group and a transition metal [2], the term “agostic” now encompasses various types of interactions [3], which are more generally defined as complexes in which the distortion of an organometallic moiety brings an appended C–H bond into close vicinity with the metal center [4]. Historically, the existence of agostic bonding was primarily inferred from structural and, less frequently, spectroscopic considerations. Depending on the location of the interacting C–H bond relative to the metal center, agostic interactions can be classified as α, β, γ, etc. A special case of agostic complexes—anagostic complexes—are characterized by relatively long M⋅⋅⋅H distances (≈2.3–2.9 Å) and large M⋅⋅⋅H–C bond angles (≈110–170°) [5,6,7]. In contrast, typical α-agostic bonds have shorter M⋅⋅⋅H distances (1.8–2.3 Å) and smaller M⋅⋅⋅H–C bond angles (90–130°) [3]. Aliphatic C–H α-agostic complexes are rare and quite unstable [8,9] with the metal–hydrogen interaction being the primary contributor to the bonding [10]. More stable complexes involve C-bonding with unsaturated (C_sp2_) atoms [11,12]. β-agostic complexes, the most common class of agostic complexes [3,4,13,14], are usually characterized by strong metal–C and metal–H bonding [3,13]. The formation of agostic metal–CC complexes is much rarer and typically involves at least one unsaturated (sp, sp^2^) [15,16,17,18] or highly strained C atom [1,19,20]. α,β-CCC agostic bonding has been observed in metallacyclobutanes with electron-deficient metals [14] and in metal–benzyl complexes [21].

A deeper understanding of agostic interactions necessitates the application of electronic structure methods. In quantum chemistry, a molecule is represented as electron density in the potential field of embedded nuclei. Therefore, many general chemical concepts, such as atoms and bonds, lack an unambiguous meaning and become subject to definition [22,23,24]. However, concepts such as bonding, bond order, ionicity, and electron population are still widely used to predict or explain chemical properties and reaction mechanisms [25]. In order to root these concepts in quantum chemistry, one needs to split the electron density into atomic contributions. Such partitioning can be defined in terms of atomic and molecular orbitals (Hilbert space) or by dividing the three-dimensional space of electronic charge distribution into atomic basins (real space). Techniques such as natural bond orbital (NBO) [26] and charge decomposition analysis (CDA) [27], widely used to characterize the nature of agostic bonding, are based on wave function (MO) analysis. More recently, agostic bonding has been examined using the quantum theory of atoms in molecules (QTAIM) [28] and electron localization function (ELF) [29,30,31] methods, which analyze the topology of electron density and provide a physically sound basis for defining atoms and bonds in a molecule [32]. Different partitioning schemes may lead to qualitatively different conclusions [33].

Depending on the number of electrons involved in metal–H–C bonding, hydrogen bonds are three-center-four-electron (3c–4e), agostic bonds are three-center-two-electron (3c–2e), while anagostic bonds are largely electrostatic with no electron sharing [34,35]. The delocalization of C–H bonding electrons into a vacant orbital on the metal is identified as the major driving force for agostic bonding in electron-deficient metals [34]. The existence of a vacant MO on the metal is a mandatory condition for agostic interaction in these complexes [36,37]. Agostic bonding in late transition-metal complexes is traditionally described in terms of the Dewar–Chatt–Duncanson (DCD) model [38,39,40,41]. Recent computational studies showed that noncovalent interactions play an extremely important role in agostic interaction [42,43]. Thus, the DLPNO-CCSD(T) calculations by Neese et al., followed by energy decomposition, showed that London dispersion forces are key driving forces in agostic complexes [44,45]. The significant role of hyperconjugation was highlighted by Lin et al. based on VB studies [46]. Thus, the nature of agostic bonding requires further elucidation [13,19] and the development of a common view based on different approximations.

**Scheme 1:**
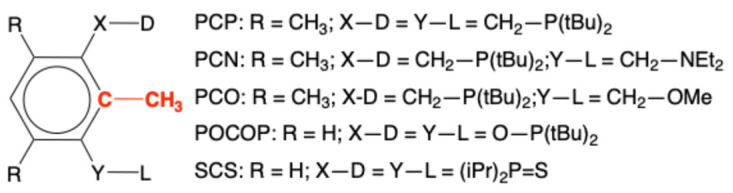
Pincer ligands studied in the present work.

Rhodium (I) complexes with pincer ligands exhibit various types of agostic interactions [47]. The simplest and weakest bonding that allows hydrocarbon coordination is anagostic Rh–H–C_sp3_ interaction [48]. Aromatic hydrocarbons tend to form agostic Rh–C_sp_^2^–H complexes [39,49], featuring both CH → metal donation and backdonation, resulting in C–H bond activation. However, κ^1^–H and κ^1^–C bonding patterns are also found in metal–C_sp2_H interactions in pincer complexes [50,51,52,53,54]. While investigating the reaction between the cationic Rh(I) precursor [Rh(cyclooctene)_2_(acetone)_2_]BF_4_ and a thiophosphoryl-based, SCS-type pincer ligand, computational evidence was found for a reaction intermediate featuring a novel η^3^–C–C–H agostic interaction [39]. This intermediate serves as a common entry point to both C*_ipso_*−C_Me_ bond cleavage, observed experimentally, and C_sp3_−H bond cleavage, which was not observed [55]. Reexamining the detailed mechanism of C_sp2_–C_sp3_ and C_sp3_–H oxidative addition in tridentate Rh(I) complexes with different pincer ligands, shown in Scheme 1, revealed that a common η^3^-agosticagostic intermediate precedes both cleavage reactions in all these systems [56]. The architecture of these complexes, with an arene backbone, two ligating side-arms in meta positions, and an alkyl moiety situated between those arms, keeps both C_sp2_–C_sp3_ and C_sp3_–H bonds in close proximity to the metal center. However, the nature of bonding in such specific agostic structures is not fully understood. In this work, we examine the nature of bonding in the η^3^-agostic Rh(I) complexes with and without ancillary ligands using wave function and electron density analyses. Such a combined approach is often applied to interpret complex bonding situations [50,57,58,59,60].

## 2. Results

### 2.1. Geometries of the η^3^-Agostic Complexes

**Figure 1:**
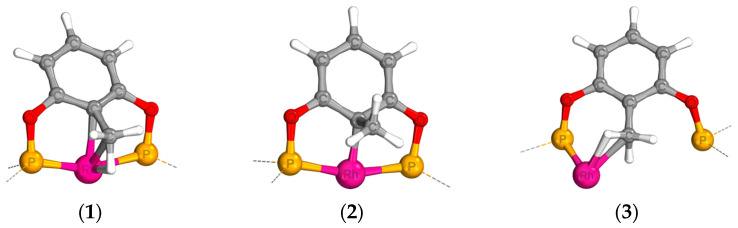
Typical types of precleavage pincer complexes, as represented by the POCOP-Rh system. Hydrogen atoms and alkyl groups not involved in bonding with Rh have been omitted for clarity. The exact structure of the POCOP pincer ligand is detailed in Scheme 1. (**1**) η^3^-C*_ipso_*–C_Me_−H agostic (**2**) κ–C*_ipso_* (**3**) η^2^-C_Me_–H agostic.

The typical structure of the Rh(I) η^3^-C*_ipso_*–C_Me_−H agostic complex (**1**) is representatively shown in Figure 1 for the Rh–POCOP system. Alongside the η^3^-agostic complexes, precleavage complexes with nearly identical free energy, characterized by metal–arene interactions in a κ–C*_ipso_* (or η^2^-C*_ipso_*–C*_ortho_*) configuration with no involvement of the methyl group (**2**) and notably less stable η^2^-C_Me_−H agostic complexes with no participation of the arene ring (**3**) could be found as local minima in several systems, especially in the absence of ancillary ligands [56]. In the absence of ancillary ligands, η^3^-agostic complexes are often stabilized by additional Rh bonding with an H atom of the *p*-*t*Bu arm. Selected optimized geometric parameters of the various η^3^-agostic pincer complexes in the absence and in the presence of ancillary ligands are compiled in Table S1 in the Supplementary Materials. In the absence of ancillary ligands, the Rh–H bond length in these complexes varies from 1.66 Å in Rh–SCS to 1.91 Å in Rh–PCN, characteristic of agostic or even for covalent bonding. Methanol coordination slightly reduces the Rh–H bond length in the PCN, PCO, and SCS complexes, yet increases it in the PCP and POCOP complexes. The coordination of electron-withdrawing ligands, ethylene, and CO, elongates the Rh–H bond by 0.11–0.38 and 0.20–0.50 Å, respectively. The Rh–C*_ipso_* and Rh–C_sp_^3^ distances fall in the ranges of 2.16–2.42 and 2.10–2.63 Å, respectively, in comparison with the typical covalent Rh–C_sp2_ and Rh–C_sp_^3^ bond lengths of 2.04 and 2.11 Å [61] and η^2^ CC agostic bonds of 2.35–2.39 Å in the Rh–cyclopropane complexes [62]. The Rh–C_sp_^3^ bond lengths generally change in line with the Rh–H distances upon coordination of the ancillary ligands, whereas Rh–C*_ipso_* bond lengths change in the opposite direction in the presence of methanol and show much smaller and less systematic response to the presence of electron-withdrawing ligands. Thus, C_2_H_4_ and CO ligands increase the Rh–C_sp_^3^ bond length by 0.17–0.44 Å, whereas the Rh–C*_ipso_* bond length increases by up to 0.24 Å, and in several systems, it even slightly decreases.

The bond angles Rh–C*_ipso_*–C_sp3_ (ranging from 64.0° in Rh–PCP to 83.9° in Rh–PCO–CO) and Rh–C_sp3_–H (from 49.1° in Rh–PCP to 57.4° in Rh–POCOP–MeOH) suggest the formation of agostic structures. In most complexes (except for Rh–PCO–MeOH, Rh–POCOP–MeOH, and Rh–SCS–C_2_H_4_), the dihedral angles Rh–C*_ipso_*–C_sp3_–H show only minor deviation from planarity.

### 2.2. Electron Density Analysis

#### 2.2.1. NCIPLOT

NCIPLOT approach [63,64,65] is a tool that allows for the visualization and qualitative representation of non-covalent interactions (NCI). It is based on the analysis of the reduced density gradient, derived from the density ρ(r) and its first derivative ∇ρ(r). This method is a good starting point to probe the nature of agostic bonding since it allows us to identify and analyze unexpected NCIs, such as weak ionic bonds, hydrogen bonds, and van der Waals interactions (so-called closed-shell interactions), in which ∇ρ(r) ≠ 0.

**Figure 2:**
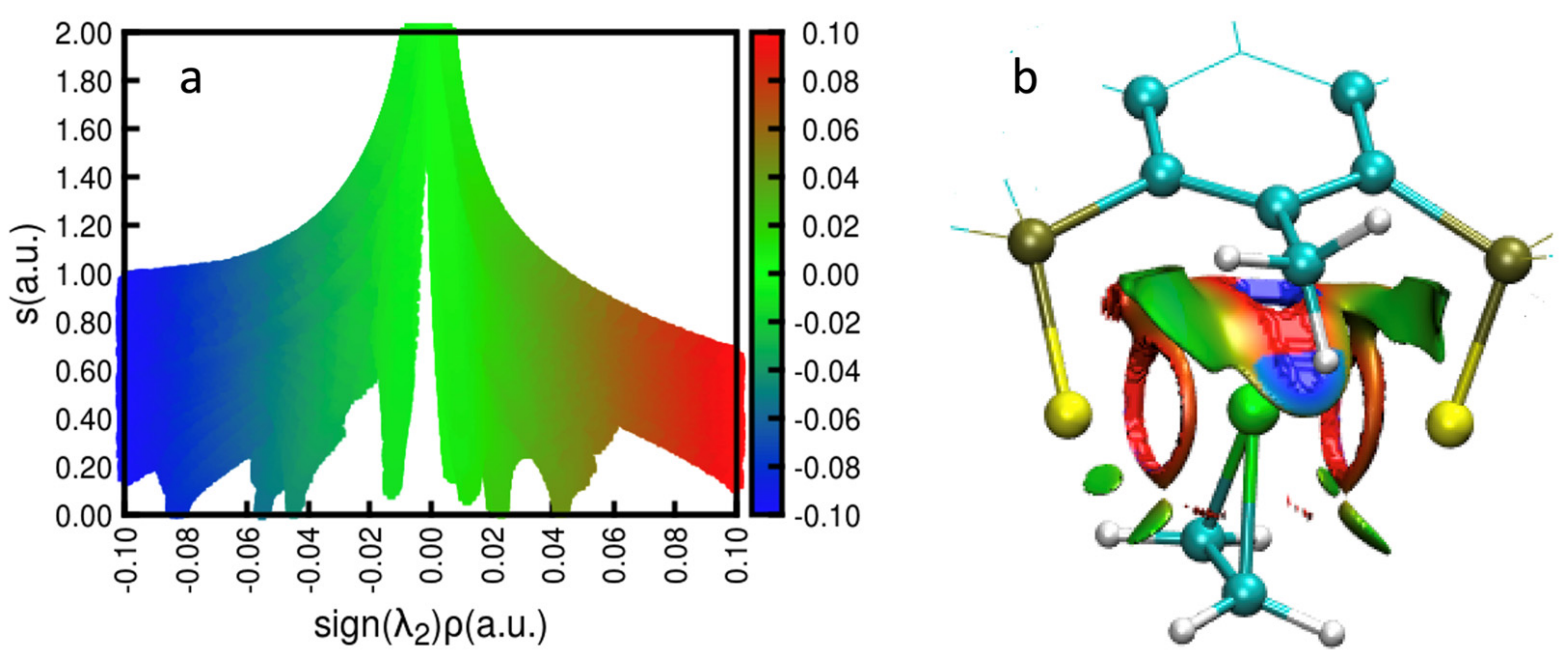
Two-dimensional NCI plot of the reduced density gradient versus the electron density multiplied by the sign of the second Hessian eigenvalue (**a**) and gradient isosurfaces (**b**) for Rh–SCS–C_2_H_4_ complex (s^pro^ = 0.5 with a ρ(r) = 0.10 cutoff). The colors are on a blue–green–red scale according to values of sign(λ_2_)ρ. The electron density was analyzed within a radius of 3.0 Å around Rh atom. Hydrogen atoms and alkyl groups not involved in bonding with Rh have been omitted for clarity. The exact structure of the SCS pincer ligand is shown in Scheme 1.

The 2D plots of the reduced density gradient versus the electron density multiplied by the sign of the second Hessian eigenvalue are shown in Figure 2a and Figure S1 in the Supplementary Materials. These plots display the existence of strong attractive (blue), strong repulsive (red), and weak (green) noncovalent interactions, each represented by several spikes in the corresponding area. The visual representation of 3D isosurfaces (Figure 2b and Figure S1) reveals that various interactions, including van der Waals interactions with the methyl groups of the pincer ligand arms, contribute to the NCI plots.

In the absence of ancillary ligands and with electron-donating methanol, most η^3^-agostic Rh complexes do not exhibit NCIs between the C_sp2_C_sp3_H group and the metal atom. In the presence of ethylene and CO, the Rh–H and Rh–C*_ipso_* interactions are attractive (colored blue) for all the complexes, whereas Rh–C_sp_^3^ interactions are mostly repulsive. The red ring of the reduced density gradient isosurface is visible around the Rh bonds with the pincer ligand arms, with the only exception being the Rh–P bond in Rh–PCO complexes. This may be attributed to π repulsion between occupied Rh d orbitals and the orbitals of the ligating atoms.

#### 2.2.2. QTAIM

QTAIM analysis provides a rigorous mathematical framework to define atoms and bonds within a molecule, focusing on the properties of critical points on the electron density, ρ(r), where ∇ρ(r) = 0, i.e., the points with an NCI index s(ρ) = 0. The bonding nature in QTAIM is mainly inferred from bond critical points (*bcp*) and ring critical points (*rcp*). The *bcp*, or (3,−1) critical point, is located along the path of maximum electron density, which connects two nuclei and represents the point where the electron density reaches a minimum along the bond path but is maximal in the perpendicular directions. At the *bcp*, the Laplacian of electron density ∇^2^ρ is positive for closed-shell interactions and negative for covalent bonds. These critical points are typically surrounded by low-valued isosurfaces, analyzed by NCIPLOT. The *rcp*, or (3,+1) critical point, represents a saddle point with the minimum electron density within the ring surface and a maximum in the perpendicular direction. The term “bond critical point” has traditionally been used in QTAIM. However, recent studies suggest that bond paths and *bcp*s can appear in situations where chemical bonding is not expected based on fundamental chemical concepts, nor is it confirmed by experimental or other theoretical methods [66,67,68,69]. Conversely, a *bcp* may be absent even in cases where significant electron density sharing between two atoms is observed [70,71,72]. For this reason, the term “line critical point” (*lcp*) is being increasingly recommended in the literature [73,74]. In this manuscript, the terms “bond critical point (*bcp*)” and “line critical point (*lcp*)” are used interchangeably, both referring to the same (3,−1) critical point. Similarly, “bond path” is used interchangeably with “line path”. The relationship between bonding in η^3^-agostic complexes and the presence of the line path and *lcp* will be discussed in detail.

The atomic charges on Rh in the η^3^-agostic Rh pincer complexes (Table S2) show a total charge transfer from the pincer ligands to the Rh(I) cation, ranging from 0.54 to 0.84 electrons. The electron donation strength decreases in the following order: POCOP > PCP > PCO > PCN > SCS. The transferred electron density primarily comes from P atoms of PCP, PCN, and PCO, from S atoms of SCS, and from the aromatic ring of the POCOP ligand. The C*_ipso_*, C_Me,_ and H_Me_ atoms acquire up to 0.07, 0.10, and 0.04 electrons, respectively, upon bonding with Rh. In only the PCO and SCS complexes, H_Me_ atoms are slightly more positively charged than in the free pincer ligands.

**Figure 3:**
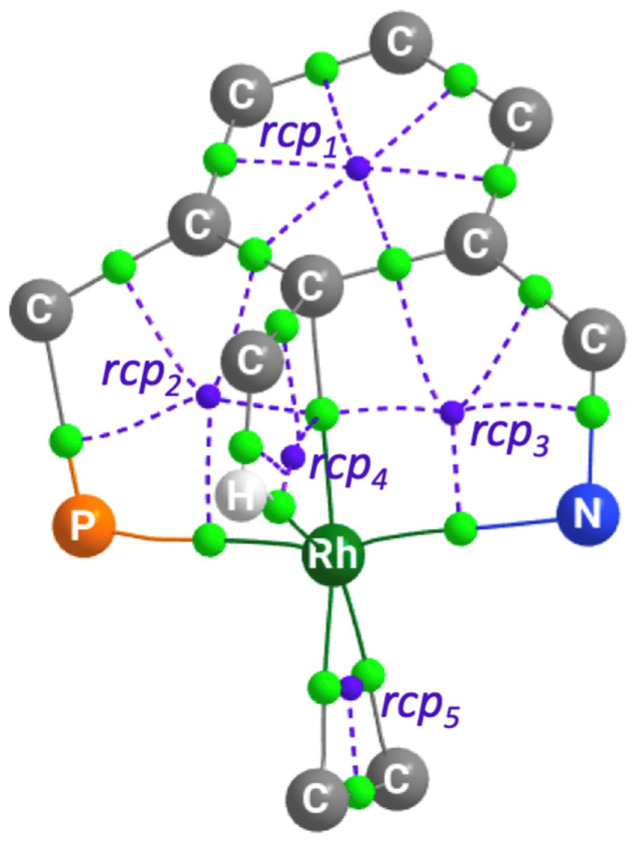
Typical molecular graph of η^3^-agostic complex Rh–PCN–C_2_H_4_ showing line paths (solid atom-colored lines) with line critical points (small green spheres) and ring paths (dashed blue lines) with ring critical points (*rcp*, small blue spheres). Hydrogen atoms and alkyl groups not involved in bonding with Rh are omitted for clarity. The exact structure of the PCN pincer ligand is shown in Scheme 1.

Previous QTAIM studies of agostic late transition metal alkyl complexes revealed only two *bcp*s, indicating Rh–C*_ipso_* and Rh–H bonds, with an absence of Rh–C_Me_ bonding [38]. This absence was attributed to the atomic basins of the C*_ipso_* and H_Me_ atoms spreading around the Rh basin and inhibiting the formation of a *bcp* between Rh and C_Me_. The molecular graphs obtained in our calculations (Figure 3 and Figure S2) typically display *bcp*s for two Rh bonds with the ligand arms, Rh–C*_ipso_*, Rh–H, and Rh bonds with possible ancillary ligands, while Rh–C_Me_ *bcp*s are absent in all studied η^3^-agostic complexes. An exception is the Rh–POCOP–MeOH complex, which does not involve any Rh–H bonding, and several complexes (Rh–PCP, Rh–PCO, Rh–PCO–MeOH, and Rh–SCS–MeOH) do not exhibit the Rh–C*_ipso_ bcp*.

**Figure 4:**
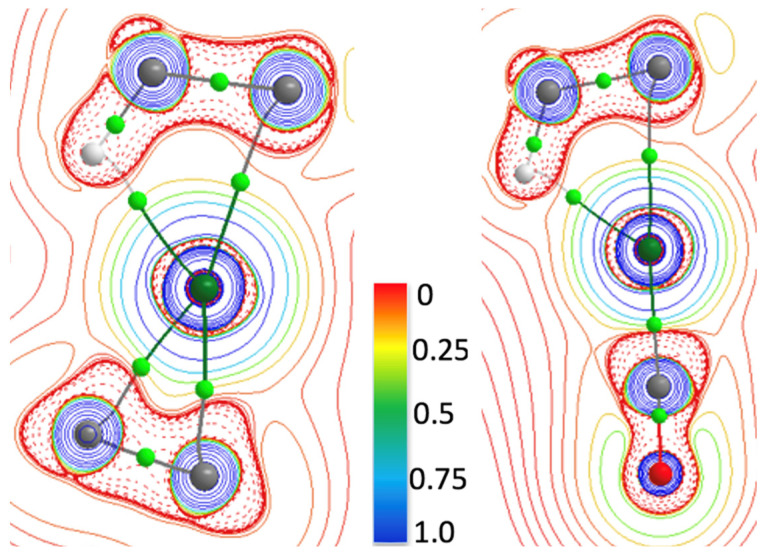
Typical Laplacian of the electron density distribution in η^3^ Rh–PCP complexes with ethylene (**left**) and CO (**right**) in the Rh–C*_ipso_*–C_Me_ plane. Dashed lines represent negative values.

Phosphorus atoms of the ligand arms form the strongest bonds with Rh, indicated by low potential energy density (V) and high electron delocalization index (DI) in Table S3, while S and O atoms are bound much weaker. The contribution of the bond between Rh and the ligand arms to the topological charge on Rh (q(A|B) values in Table S3) shows that P atoms act as electron donors, while more electronegative N, S, and O atoms have an electron-withdrawing effect. The strongest Rh–C*_ipso_* bonds are found in Rh–SCS and Rh–POCOP–MeOH complexes. The charge flow in Rh–C*_ipso_* bond streams from Rh to the aromatic ring; therefore, π acceptor ligands C_2_H_4_ and CO weaken the Rh–C*_ipso_* interaction. The electron density at the Rh–H *lcp* ranges from 0.04 to 0.12 a.u., slightly higher than usual for agostic bonds (0.04–0.05 a.u.). The Laplacian of electron density (0.13–0.27 a.u.) matches the expected range for agostic bonding (0.15–0.25 a.u.) [28]. The positive Laplacian values indicate closed-shell interactions between Rh and all the ligands (Figure 4, Figure S3 and Table S3).

**Figure 5:**
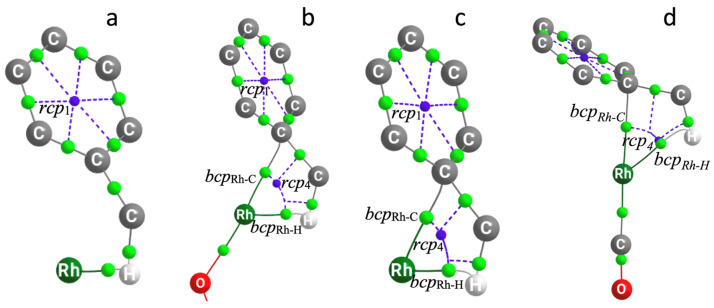
Molecular graphs illustrating the bonding patterns of the η^3^-agostic moiety in Rh–PCP (**a**), Rh–PCP–MeOH (**b**), Rh–POCOP (**c**), and Rh–PCO–CO (**d**) η^3^-agostic complexes.

Both C–H and C–C bonds weaken upon the formation of η^3^-agostic complexes (more positive V and smaller DI, Table S3). The CO and ethylene ligands destabilize Rh bonds with the pincer ligands in the η^3^-agostic complexes while methanol slightly stabilizes Rh–H bonding. The only exception is the Rh–POCOP complex, in which methanol coordination facilitates Rh–C*_ipso_* bonding on the penalty of Rh–H bond breaking. The Rh–H line path lengths (LPL) are notably longer than the geometric path lengths (GPL) (by up to 0.21 Å in the Rh–PCO–CO complex). The curvature of the line paths is in proximity to the hydrogen atom, whereas the *lcp*-Rh part of the line paths is straight (Figure 5b–d). Together with slightly positive charges on the hydrogen atoms, it excludes the hydride nature of the Rh–H bond [75,76,77].

The ring critical point (*rcp*) is a saddle-shaped critical point that is opposite to the *lcp*. The close proximity of the *rcp* and *lcp* serves as an indicator of structural instability within the molecular structure, for even a slight geometric perturbation can lead to the merger and nullification of the *rcp* and *lcp* [28,78,79]. In Figure 3, an assortment of *rcp* types observed in the η^3^-agostic complexes is illustrated. Specifically, *rcp*_1_ corresponds to a six-membered aromatic ring, whereas the Rh atom partakes in the generation of five-membered rings in conjunction with the phenyl and ligand arms P/S (*rcp*_2_) and P/S/N/O (*rcp*_3_). A distinctive hallmark of η^3^-agostic bonding is the presence of a four-membered ring, represented by *rcp*_4_. Additionally, an ethylene ligand forms a three-membered ring with Rh, denoted by *rcp*_5_. Selected QTAIM properties of these *rcp*s in the η^3^-agostic Rh complexes are collected in Table S4.

The distances between *rcp* and its neighboring Rh–P, as well as Rh–C*_ipso_ bcp*s within the five-membered rings, encompass a range of 1.1 to 1.2 Å (Table S5). The distances to the Rh–S *bcp* are marginally greater, extending up to 1.34 Å. In contrast, distances to Rh–N and Rh–O *bcp*’s exhibit diminution, approximating 1.0 Å and 0.9 Å, respectively, in agreement with the Rh–N and, especially, Rh–O arm opening frequently observed in experiments. The separation between *rcp*_4_ and its adjacent Rh–C and Rh–H bond critical points is notably shorter (Table 1 and Table S5). The *rcp*_4_-*bcp*_Rh–H_ distance in Rh–PCO–CO is as small as 0.01 Å; in several complexes *bcp* and *rcp* coalesce, and the agostic ring structure disappears. The position of *rcp*_4_ relative to *bcp*_Rh–C_ and *bcp*_Rh–H_ is controlled by pincer and ancillary ligands. For instance, *rcp*_4_ is located closer to *bcp*_Rh–H_ in Rh–PCN complexes in the absence of ancillary ligands and in the presence of electron-withdrawing ethylene and CO. However, the presence of methanol prompts its migration towards *bcp*_Rh–C_. In Rh–POCOP, *rcp*_4_ is in proximity to the Rh–C bond critical point; its relocation in the vicinity of *bcp*_Rh–H_ is observed in the presence of C_2_H_4_ and CO, whereas the coordination of an electron-donating MeOH leads to the disappearance of *bcp*_Rh–H_. Illustrative examples are shown in Figure 5. The importance of structural instability for understanding the nature of bonding in η^3^-agostic complexes will be discussed below.

#### 2.2.3. Electron Sharing Indices in 3D (ESI-3D)

The sharing of electron density is the primary force that binds atoms together. Consequently, electron sharing indexes (ESI) and the closely related concept of bond order are naturally used as a measure of bond strength. Various computational schemes based on different atomic partitions have been suggested, tracing back to the works of Coulson [80], Mulliken [81], Mayer [82,83,84,85], and Wiberg [86]. Most of these schemes rely on Hilbert space partitioning and encounter drawbacks when applied with correlated computational methods and large basis sets. An alternative approach involves the spatial atomic partition of exchange-correlation density using, for example, schemes of Bader [87] or Hirshfeld [88]. In this study, we employed the ESI-3D approach of Matito et al. [89,90,91] to determine bond orders of regular and multicenter bonds from the real-space QTAIM partitioning of electron density. In this scheme, two-center indexes (2c-ESI) coincide with delocalization indexes DI in Bader’s theory, provided a line critical point (*lcp*) exists between the two atoms. Thus, ESI-3D allows the examination of electron delocalization between atoms that lack a line path in QTAIM as well as among groups of three or more atoms. The ESI-3D method was recently used to distinguish between three-center (3c) agostic, anagostic, and hydrogen bonds [35]. Hydrogen bonds were found to have 3c-ESI values lower than 0.005, anagostic interactions between 0.005 and 0.040, and agostic interactions had 3c-ESI values higher than 0.040. It was demonstrated that *bcp*s are absent for complexes with 3c-ESI values in the range of 0.025–0.050 [35].

Our calculated 4c-ESIs for the Rh–C*_ipso_*–C_Me_–H ring and 3c-ESIs for the Rh–C*_ipso_*–H (β-agostic) bond are negligibly small, indicating the absence of cyclic electron delocalization in the η^3^-agostic rhodium complexes (Table S6). On the other hand, three-center indexes for Rh–C*_ipso_*–C_Me_ (0.033–0.075) and Rh–C_Me_–H (0.045–0.112) bonds fall into the range of agostic bonding [27]. Only two complexes, Rh–PCN–CO and Rh–PCO–CO, display Rh–C_Me_–H bonding in the anagostic range with 3c-ESIs of 0.001 and 0.037, respectively. The two-center indexes indicate the quite strong bonding of C*_ipso_*, C_Me_, and H atoms with Rh (2c-ESIs vary in the ranges of 0.266–0.636, 0.090–0.547, and 0.128–0.462, respectively). Notably, all three atoms show comparable 2c-ESI values. The only complex for which the Rh–H interaction is much weaker (2c-ESI = 0.051) is Rh–POCOP–MeOH, the same complex that lacked Rh–H interaction in the QTAIM analysis (*vide supra*). Complexes for which the Rh–C*_ipso_* bond is absent in QTAIM do not necessarily have small 2c-ESI values for this bond. Thus, ESI-3D calculations represent the η^3^-agostic bonding as two η^2^ agostic units, Rh–C*_ipso_*–C_Me_ and Rh–C_Me_–H, in contrast to QTAIM showing formally β-agostic C*_ipso_*–H bonding in most complexes.

### 2.3. Molecular Orbital (MO) Analysis

#### 2.3.1. NBO

Natural bond orbital (NBO) analysis is one of the most widely used methods for interpreting many-electron wavefunctions in terms of chemical bonding concepts [92,93,94,95]. NBO analysis breaks down the electron density in a molecule into electron-pair bonding units derived from an idealized Lewis structure. In the context of non-covalent forces, NBO analysis employs second-order perturbation theory to quantify the strength of donor-acceptor interactions between occupied and virtual orbitals. Natural population analysis (NPA), based on the atomic decomposition of natural bond orbitals, allows for the evaluation of atomic charges.

NPA charges on selected atoms and groups in the η^3^-agostic Rh(I) complexes are collected in Table S7. The charges on the ligating atoms of the uncoordinated pincer ligands qualitatively agree with the QTAIM results; *p*-atoms are strongly positively charged, S, N, and O atoms maintain negative charges, and C*_ipso_* atoms are nearly neutral. The C–H bonds in the methyl group are represented as ionic, with atomic charges of −1.0 and +0.3 on the C and H atoms, respectively, contrasting with QTAIM results, which show negligible charges on these atoms (Table S2). Agostic bonding decreases the positive charge on hydrogen by about 0.1 electron and simultaneously reduces the negative charge on C_Me_ by about 0.35 electron. In contrast, a significant positive NBO atomic charge of the agostic hydrogen atom is distinctive for η^2^ agostic complexes [96]. The Rh atomic charges in the η^3^-agostic complexes show even stronger deviations between QTAIM and NBO results: in the former case, they range from 0.16 to 0.48, while in the latter case, Rh atoms often exhibit negative charges of up to −0.25. Moreover, compared to QTAIM, NPA overestimates the donation ability of ancillary ligands. For example, CO appears to be as strong an electron donor as methanol, and ethylene shows virtually zero total electron transfer, whereas both ligands maintain negative charges, with CO being a stronger withdrawing group than ethylene in QTAIM. Despite such significant differences, both computational schemes show similar alterations in atomic charges across most of the series of similar complexes.

The calculated Wiberg bond indexes (WBI) indicate a slight weakening of the C_sp2_– C_sp3_ bond and a notable weakening of the C_sp3_–H bond upon the formation of most η^3^-agostic complexes (Table S8). The strongest activation was observed in the Rh–SCS–MeOH complex, where the C–H bond order drops to 0.50 compared to 0.91 in free SCS. Significant WBIs were found for the Rh–C*_ipso_* and Rh–H bonds in most complexes. The largest values of 0.63 and 0.40, respectively, were found in Rh–SCS–MeOH. The smallest Rh–H bond order of 0.04 was found in Rh–POCOP–MeOH, the only complex lacking the Rh–H bond critical point (*bcp*) in QTAIM. However, several other complexes (Rh–PCO–CO, Rh–PCN–CO, and Rh–POCOP–C_2_H_4_) had similarly small Rh–H bond orders. The smallest Rh–C*_ipso_* WBI of 0.09 was found in Rh–PCP, the complex that lacks the Rh–C*_ipso_ bcp* in QTAIM. However, other complexes without an Rh–C*_ipso_ bcp* have quite large WBI values, including Rh–SCS–MeOH with the largest Rh–C*_ipso_* bond order. The NBO results correlate better with ESI-3D than with QTAIM particularly, showing significant Rh–C_Me_ bond orders (0.09–0.32).

Lewis-like transition metal complexes tend to form hypervalent bonds [97]. Indeed, in the PCP, POCOP, and SCS complexes, NBO analysis reveals the presence of three-center, four-electron P/S-Rh–P/S bonds, where bonding Rh–P/S interactions are accompanied by antibonding orbitals composed of occupied Rh 4d_x2-y2_5s and P/S 3s3p_x_ atomic orbitals (with Rh–P/S bonds oriented close to the *x*-axis direction). For example, in the Rh–SCS–C_2_H_4_ complex shown in Figure 2b, this antibonding orbital has an occupancy of 0.46 electrons. These interactions likely account for the red rings surrounding the Rh–P/S bonds in the NCIPLOT.

Neither covalent nor three-center bonds between the Rh atom and any C_sp3_–H or C_sp2_–C_sp3_ bond were found by NBO analysis. However, second-order perturbative analysis in the NBO basis reveals numerous dative interactions of Rh with C–C and C–H bonds, as well as with individual C_sp2_, C_sp3_, and H atoms of the η^3^-agostic moiety (Table S9). For instance, the dative interaction in the Rh–SCS complex consists of σ_C–C_ → Rh 5s and σ_C–H_ → Rh 5s stabilizing contributions of 11.46 and 45.27 kcal/mol, respectively. Additionally, there are weaker (0.6–1.1 kcal/mol) donations from σ_C–C_ to virtual Rh 5p and 5d orbitals and numerous donations from σ_C–H_ to the Rh virtual orbitals (up to 11 kcal/mol in strength), resulting in a total stabilization from C–C and C–H donations estimated to be 16.13 and 130.88 kcal/mol, respectively. Charge transfer in the opposite direction includes two Rh 4d → σ*_C–C_ interactions with stabilization energies of 0.68 and 4.50 kcal/mol and two Rh 4d → σ*C–H interactions contributing 20.74 and 3.04 kcal/mol. Furthermore, NBO analysis reveals backdonation from Rh to each atom of the η^3^ moiety. Similar interactions were found in most studied complexes. In the presence of ancillary ligands, these contributions tend to diminish, but additional dative interactions appear in several complexes. Thus, σ_C–C_ → Rh donation disappears, and σ_C–H_ → Rh significantly drops in all carbonyl complexes, and this electron density is mostly transferred to σ*_Rh–C(CO)_ orbitals. A more complex picture was observed in the presence of an ethylene ligand. The best Lewis structures found for Rh–PCP–C_2_H_4_ and Rh–SCS–C_2_H_4_ are single units with notable σ_C–C_ → σ*_Rh–C_(C_2_H_4_)__ (6.7 kcal/mol), σ_C–H_ → σ*_Rh–C_(C_2_H_4_)__ (21.3–22.2 kcal/mol), and Rh–C_C_2_H_4__ → σ*_C–H_ (7.8 kcal/mol) stabilizing interactions. For the other three complexes, the Lewis structures found by NBO analysis treat the ethylene ligand as an independent unit with no interaction with the η^3^-agostic unit. To maintain consistency in analyzing the electronic structure of the olefinic complexes, the ethylene ligand was defined as an independent unit in Rh–PCP–C_2_H_4_ (Table S9). However, we were unable to determine a reasonable Lewis structure for the Rh–SCS–C_2_H_4_ complex with an independent ethylene unit. For the other complexes, these Lewis structures exhibit minor stabilization from donation (3.5–6.1 kcal/mol) and backdonation (1.1–6.2 kcal/mol) between Rh and C–C bonds, significant stabilization by Rh → C_ipso_ interactions (20.0–27.6 kcal/mol), and, in most cases, by σ_C–H_ → Rh electron transfers (2.4–18.2 kcal/mol). Nevertheless, the primary stabilization in these complexes stems from the exceptionally strong π_C–C_ → Rh and Rh → π*_C–C_ interactions between Rh and ethylene (74.4–217.1 and 67.2–82.4 kcal/mol, respectively).

One major issue with NBO analysis is that it often fails to find a correct Lewis structure for transition metal complexes with unusual bonding. In a proper natural Lewis structure, bonding (Lewis) orbitals with two-electron occupancy are complemented by formally empty antibonding (non-Lewis) orbitals. Occupancies of the antibonding orbitals reveal deviations from an idealized localized Lewis structure. Noncovalent agostic interactions are inherently inconsistent with traditional Lewis structures, making NBO tools less applicable to agostic complexes. We found up to 0.4 electron holes in the Lewis orbitals and more than 0.5 electron occupancy of several antibonding orbitals. This leads to unrealistic negative charges on Rh(I) in some complexes (Table S7) and extremely high energy terms in the second-order perturbation theory analysis (Table S9). Energy values higher than 50 kcal/mol (highlighted in Table S9) suggest that Lewis structures do not accurately describe the density matrix. These findings clearly show that NBO analysis is not very effective for interpreting the electronic structure of η^3^-agostic complexes.

#### 2.3.2. IBO

The Intrinsic bond orbitals (IBO) scheme by Knezia et al. [98,99] employs an intrinsic minimal basis to accurately represent the occupied molecular orbitals (MOs) of a previously computed SCF wave function. This method does not rely on the molecule’s Lewis structure and is mostly free from empirical input. It enables atomic partitioning in Hilbert space, unaffected by the basis set, and adheres to trends in electronegativities. This approach clearly differentiates between σ and π orbitals and has been effectively used to analyze bonding in terms of donor–acceptor interactions [100].

The calculated IBO atomic charges (Table S9) in the η^3^-agostic Rh complexes show a qualitative correlation with the topological charges (Table S2). Quantitatively, IBO and QTAIM charges may differ significantly, particularly for P, N, and O atoms of the pincer ligand arms. The agostic C–H bond appears to be much more polar in the IBO scheme (with charges ranging from −0.387 to −0.438 on C_sp3_ and from 0.109 to 0.157 on H), whereas QTAIM finds the C–H bond almost nonpolar. Wiberg bond indexes (WBI) on the IBO basis (Table S10) exhibit similar qualitative trends to the ESI-3D two-center indexes. Thus, all three atoms of the agostic moiety are bonded to Rh(I), with WBI values falling in the range 0.109–0.379 for Rh–C_sp2_, 0.047–0.354 for Rh–C_sp_^3^, and 0.037–0.279 for Rh–H. Moreover, both methods indicate that the Rh–C_sp_^3^ bond is stronger than the Rh–C_sp2_ bond in several complexes, especially in the absence of ancillary ligands and in the presence of methanol. Quantitatively, the WBI values in the IBO basis are 0.1–0.24 smaller than the corresponding ESI 2c indexes.

Analysis of the intrinsic bond orbitals (Table 2) reveals both σ donation and π backdonation between Rh and the pincer ligand arms. Weaker electron donation from the highly electronegative O arm of the PCO ligand is compensated by increased P→Rh electron transfer in Rh–PCO. Some release of electron density occurs due to Rh→P backdonation, whereas N, O, and S atoms show no electron-withdrawing ability. The IBO analysis supports the η^3^ nature of the complexes (Table 2). Indeed, weak mixing of the Rh d orbitals with the occupied C*_ipso_*–C_Me_ and C_Me_–H σ orbitals, which have comparable contributions from C*_ipso_*, C_Me_, and H atoms, was observed in all η^3^ complexes. Additionally, all three atoms participate in weak mixing with occupied Rh d orbitals in all the complexes except for PCN. Furthermore, some phenyl → Rh electron transfer was identified in the systems showing Rh–C*_ipso_* bonding in QTAIM analysis, such as Rh–POCOP, Rh–PCN, and Rh–SCS η^3^-agostic complexes.

The key orbitals involved in the agostic bonding are influenced by the ancillary ligands: the ligand→Rh σ donation slightly weakens the interaction of σ C–H and σ C–C orbitals with Rh, whereas weak Rh→CCH backdonation (Table 2) that transforms into Rh→C*_ipso_* or Rh→H bonding orbitals in the dissociation products virtually disappears due to stronger Rh→C_2_H_4_ or Rh→CO π backdonation. That raises the activation barriers for both oxidative addition reactions in the presence of ethylene and CO [56].

## 3. Discussion

This paper employs a variety of computational techniques to examine the electronic structure and bonding interactions in η^3^-agostic Rh(I) complexes. It is noteworthy that different methods offer varied perspectives on the nature of η^3^-agostic bonding. The atomic charges calculated by these methods show significant differences. For instance, the QTAIM method assigns charges ranging from 0.16 to 0.48 on rhodium, while IBO assigns slightly lower charges from 0.14 to 0.40. Conversely, NBO assigns negative charges as low as −0.25 on the metal in some complexes. Among the atoms of the pincer ligand arms, P, N, and O display the largest quantitative variations in charges across these methods. For example, the nitrogen atom in the Rh–PCN agostic complex shows charges of −1.00 in QTAIM, −0.39 in NBO, and −0.21 in IBO. Regarding the C_Me_–H bonds, QTAIM suggests they are almost nonpolar, IBO indicates slight polarity (charges on C_Me_ from −0.39 to −0.44 and on H from 0.11 to 0.16), and NBO suggests high polarity (charges on C_Me_ from −0.62 to −0.67 and on H from 0.22 to 0.28).

**Figure 6:**
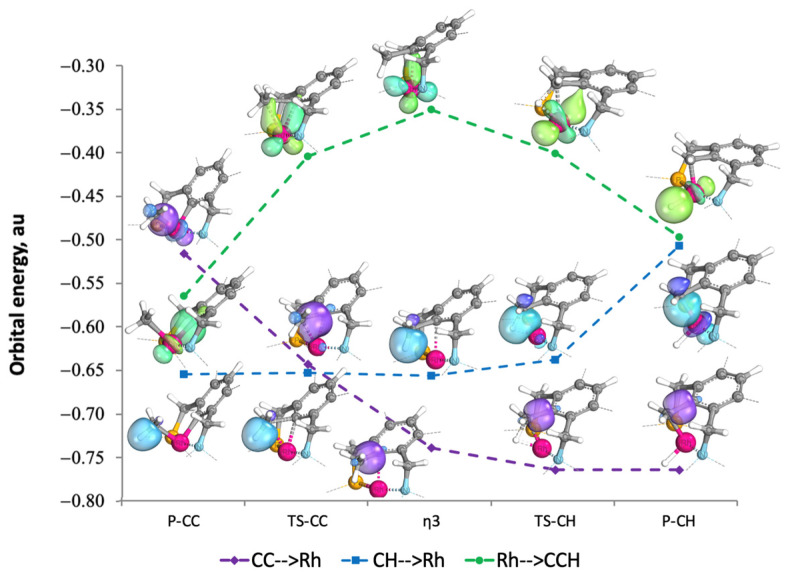
Typical changes in the energy levels of the key IBOs along the C–C and C–H bond cleavage reaction pathways in Rh–PCN systems (see Scheme 1 for the exact structure of the PCN pincer ligand).

All computational methods agree that the bonding between Rh(I) and the ligands is dative. From the geometric considerations, one might expect a four-center Rh–C*_ipso_*–C_Me_–H bonding similar to that in allyl complexes, yet calculated 4c-ESI values are negligible. In contrast, NBO, IBO, and ESI-3D analyses suggest the presence of two three-center agostic bonds: Rh–C*_ipso_*–C_Me_ and Rh–C_Me_–H, with all three atoms bonded to Rh(I). Unfortunately, the NBO method, showing non-physical negative charges on Rh(I) and high energy terms in second-order perturbation theory analysis, along with significant occupancies of antibonding orbitals indicating deviations from idealized Lewis structures, may not suitably interpret the electronic structure of η^3^-agostic complexes. IBO, proving valuable, reveals both σ donation and π backdonation between Rh and the agostic moiety. Three of these orbitals make the main contribution to the transformation of Rh(I) η^3^-agostic complexes leading to both C–C and C–H bond cleavage products (Figure 6).

**Figure 7:**
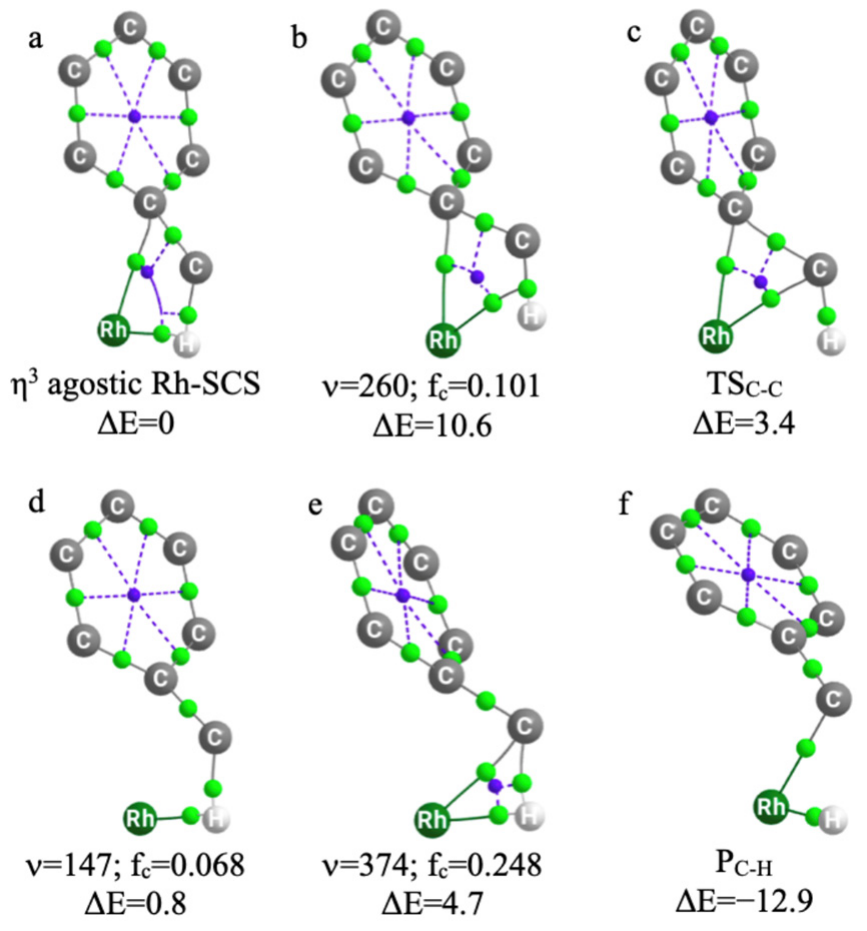
Molecular graphs of the η^3^-agostic moiety in Rh–SCS complex in the equilibrium geometry (**a**), in selected nonequilibrium geometries along low-frequency vibrational modes (**b**,**d**,**e**), in transition state for C–C bond cleavage TS_C–C_ (**c**) and in metastable C–H cleavage product P_C–H_ (**f**). Vibrational frequencies ν in cm^−1^, force constant f_c_ in mDyne/Å, relative energies ΔE with respect to the η^3^-agostic complex in the equilibrium geometry at the PBE0/TZVPP level of theory in kcal/mol.

Using QTAIM, a different portrayal emerges, highlighting the presence of Rh–C*_ipso_* and Rh–H bonds but not Rh–C_Me_. This aligns with the NCIPLOT findings of attractive densities around Rh–C*_ipso_* and Rh–H bonds and weak repulsive interactions around Rh–C_sp_^3^ bonds. Classical Bader’s theory suggests the necessity of a line path and an associated line critical point (*lcp*) for bonding. However, this one-to-one relationship has recently been questioned (see above), particularly because molecular graphs can be unstable due to zero-point nuclear vibrations [73,74]. Such instability may be seen as a sign of a weak bond appearing or disappearing with slight geometrical changes during molecular vibrations. The molecular graphs of η^3^-agostic Rh complexes, showing adjacent *lcp* and *rcp*, indicate such a dynamic instability of a line path in dynamic conditions. Following several low-frequency and low-force vibrational modes in these complexes has demonstrated the transient formation and dissolution of Rh–C*_ipso_*, Rh–C_Me_, and Rh–H line paths. Figure 5 exemplifies this dynamic, illustrating how thermal vibrations in the Rh–SCS η^3^-agostic complex cause line paths to appear and disappear. The molecular graph of the complex in its optimized geometry depicts a κ^2^–C*_ipso_*H bonding mode (Figure 7a). The low-frequency vibrational mode, with a frequency of 147 cm^−1^ and force constant of 0.068 mDyne/Å, results in the disruption of the Rh–C*_ipso_* bond (Figure 7d). The η^2^-C_Me_H bonding pattern, formed under another low-frequency and low-force vibrational mode (Figure 7e), is marked by a distinctly concave Rh–C_Me_ line path, which is 0.31 Å longer than the geometric bond distance. This shape is indicative of σ-donation from a σ-bonding orbital and backdonation into a σ*-orbital [101]. Notably, the QTAIM atomic charge on Rh in this non-equilibrium state is +0.51, compared to +0.46 in the ground state (Table S2), suggesting that σ_C–H_ to Rh and Rh to C_Me_ electron transfers might facilitate virtually non-activated C–H bond cleavage (Figure 7f). The η^2^-C*_ipso_*C_Me_ bonding pattern emerging from another low frequency and low force vibrational mode (Figure 7b) closely mimics the molecular graph of the transition state for C–C bond cleavage (Figure 7c). Similar multiple dynamic molecular graphs behavior was observed in other η^3^-agostic complexes (Figure S4). Complexes lacking Rh–C*_ipso_* or Rh–H bonds in the ground state have low-frequency and low-force vibrational modes leading to its formation.

These results lead to three key conclusions. First, although Rh–C_Me_ *lcp*s are absent in all studied complexes and the *lcp*s for Rh–C_ipso_ and Rh–H bonds vanish in several complexes at the ground state, all three bonds are readily formed and dissociated under room temperature vibrations. This suggests that there is no direct relation between the presence of *lcp* and the existence of a chemical bond in η^3^-agostic Rh complexes in equilibrium geometry. The bonding in the common intermediates leading to C–C and C–H bond cleavages can be characterized as a *dynamic* η^3^ C–C–H agostic bond. The influence of the dynamic motion on the nature of chemical bonds is now recognized in both theoretical and experimental chemistry [102,103]. Second, the kinetic preference for C–H bond cleavage is defined by the dynamic behavior of the precleavage η^3^-agostic intermediate and can be attributed to the lower reduced mass of C–H compared to C–C vibrations. Third, the energy difference between agostic and non-agostic structures, or the barrier height for methyl rotation, is traditionally viewed as an indicator of agostic bond strength [14,44]. However, our calculations show a dynamic equilibrium between Rh–C*_ipso_*, Rh–C_Me_, and Rh–H bonds, rendering such measurements of bond strength irrelevant. In fact, the breaking of the Rh–H bond is usually offset by the formation or strengthening of Rh–C bonds, leading to a minimal energy difference between the η^3^ and κ^1/2^ structures (**1** and **2** in Figure 1).

## 4. Computational Methodology

Geometry optimizations and evaluation of harmonic frequencies were carried out using the GAUSSIAN16 package [104]. All computations were performed in the gas phase utilizing the hybrid density functional PBE0 [105], and included Grimme’s empirical dispersion correction [106,107] with the Becke–Johnson damping function [108,109,110], combined with the def2-TZVPP basis set [111].

The choice of density functional is critical for accurately representing agostic bonds [112,113]. Functionals that do not adhere to the uniform electron gas (UEG) limit, particularly those based on LYP correlation, have been shown to fail in optimizing agostic structures [112,114]. Additionally, correctly describing dynamic correlation is essential. The PBE0 functional belongs to a group of functionals that effectively represent agostic bonding. Density functional theory (DFT) posits that the electron density of the ground state uniquely determines the energies, properties, and chemical behavior of molecules. However, in practice, density functional approximations (DFAs) are parameterized to reproduce energy characteristics—such as reaction and atomization energies, activation barrier heights, and weak interactions—most accurately. Consequently, a DFA’s performance in energy calculations does not necessarily translate to accurate modeling of density-based properties [115]. This issue has been extensively discussed in the literature. It has been demonstrated that, as one ascends the Jacob’s ladder of DFAs, the performance in both energy- and density-based properties generally improves, although extensively parameterized methods may show poor performance for density-based properties [116]. The hybrid PBE0 method used in this study was developed non-empirically and ranks as one of the best performers for QTAIM [112,115].

To comprehend the electronic structure and bonding in the pincer complexes discussed herein, we employed a combination of electron density and molecular orbital (MO) analyses. The NCIPLOT4 program [117] was used for the initial qualitative analysis of the electron density. A density cutoff of ρ(r) = 0.1 a.u. was applied, and the 3D images were created for an isosurface value of *s* = 0.5 and colored in the [−0.1, 0.1] a.u. sign(λ_2_)ρ range. The electron density was analyzed within a radius of 3.0 Å around Rh atom. The AIMAll program [118] was employed for calculations using quantum theory of atoms in molecules (QTAIM). The Electron Sharing Indexes Program for 3D Molecular Space Partition (ESI-3D) [89] was used to calculate two- and multi-centered bond orders in the QTAIM molecular space partition. Natural bond orbital (NBO) analysis was performed using NBO 7.0 program [119]. The IboView software version 20211019 [120] was used for intrinsic bond orbital (IBO) calculations, geometric structure, and molecular orbital (MO) representations. The IBO exponent *ρ* = 2 was used for orbital localization. The orbitals are shown as isosurfaces enclosing 80% of the orbital’s total electron density.

## 5. Conclusions

This study aimed to elucidate the detailed electronic structure and the nature of bonding in η^3^-CCH agostic rhodium complexes, which serve as a common intermediate to both C–C and C–H bond cleavages. The core findings emerged from QTAIM, ESI-3D, and IBO analyses, while NCIPLOT and NBO analyses provided additional insights. Our research established that the bonding within the η^3^-CCH agostic moiety is best described as consisting of two three-center agostic bonds: Rh–C*_ipso_*–C_Me_ and Rh–C_Me_–H, with all participating atoms forming dative bonds to Rh(I). QTAIM analysis distinctively highlights the presence of the anagostic (Rh–H) or β-agostic (Rh–C*_ipso_*–H) boding patterns in the ground state. However, thermal vibrations within these complexes can induce the formation and dissolution of bond (line) paths and bond (line) critical points for Rh–C*_ipso_*, Rh–C_Me_, and Rh–H, illustrating the dynamic nature of η^3^-agostic bonding as perceived through classical QTAIM. Collectively, the different computational techniques each contribute valuable insights, but their integrated use offers a more comprehensive understanding of the bonding dynamics in these intricate molecular systems.

## Figures and Tables

**Table 1:** Distances (Å) between ring critical point *rcp_4_* and its nearby Rh–C*_ipso_* and Rh–H bond critical points in η^3^-agostic Rh(I) complexes.

	*rcp* and *bcp*	No Ancillary Ligand	MeOH	C_2_H_4_	CO
PCP	*rcp*_4_-*bcp*_Rh–C_	-	0.430	0.522	0.486
	*rcp*_4_-*bcp*_Rh–H_	-	0.814	0.684	0.713
POCOP	*rcp*_4_-*bcp*_Rh–C_	0.505	-	0.936	0.931
	*rcp*_4_-*bcp*_Rh–H_	0.727	-	0.217	0.212
PCN	*rcp*_4_-*bcp*_Rh–C_	0.807	0.507	0.760	0.873
	*rcp*_4_-*bcp*_Rh–H_	0.354	0.772	0.478	0.292
PCO	*rcp*_4_-*bcp*_Rh–C_	-	-	0.734	0.981
	*rcp*_4_-*bcp*_Rh–H_	-	-	0.459	0.098
SCS	*rcp*_4_-*bcp*_Rh–C_	0.310	-	0.749	0.266
	*rcp*_4_-*bcp*_Rh–H_	0.817	-	0.414	0.892

**Table 2:** Composition of intrinsic bond orbitals (IBO) responsible for dative interactions in η^3^-agostic Rh(I) complexes in the absence of ancillary ligands.

	*P/S* *→* *Rh*	Rh	P/S		*P/N/O/S* *→* *Rh*	Rh	P/N/O/S	
PCP	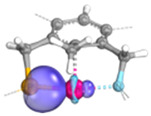	0.50	1.44		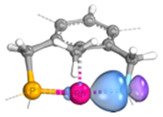	0.33	1.63 ^a^	
POCOP		0.52	1.44			0.31	1.66 ^a^	
PCN		0.52	1.45			0.22	1.70 ^b^	
PCO		0.70	1.28			0.09	1.88 ^b^	
SCS		0.38	1.59 ^a,b^			0.37	1.59 ^b^	
								
	*Rh→P/S*				*Rh→P/N/O/S*			
PCP	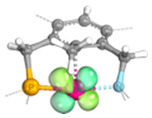	1.90	0.04		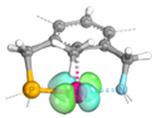	1.90	-	
POCOP		1.91	0.02			1.87	0.08 ^a^	
PCN		1.94	0.02			1.92	- ^b^	
PCO		1.91	0.05			1.94	-	
SCS		1.99	-			1.95	-	

	*C–C→Rh*	Rh	C*_ipso_*	C_Me_	*C–H→Rh*	Rh	C_Me_	H_Me_
PCP	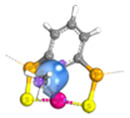	0.02	0.95	1.01	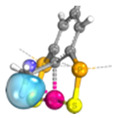	0.19	0.98	0.79
POCOP		0.05	0.96	0.97		0.11	1.03	0.83
PCN		0.05	0.96	0.98		0.08	1.07	0.83
PCO		0.03	0.95	1.01		0.17	0.99 ^d^	0.79
SCS		0.15	0.91	0.92		0.08	1.11	0.78
								
	*Rh→CCH*				*Rh→CH*			
PCP	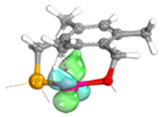	1.93	0.03	-	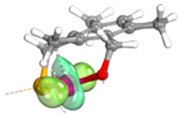	1.86	0.03	0.06
POCOP		1.85	0.12 ^c^	-		1.93	0.02	0.03
PCN		1.82	0.09	-		1.96	-	-
PCO		1.83	0.06 ^e^	-		1.92	0.03	0.03
SCS		1.54	0.33	0.07		1.96	-	0.02

^a^ Total for two P/S atoms with small contribution (~0.04) from the second atoms. ^b^ Small contribution (up to 0.04) from P atoms. ^c^ Total contribution of the phenyl group. ^d^ Small contribution (0.02) of the C*_ipso_* atom. ^e^ Small contribution (0.03) from the agostic H atom.

## Data Availability

Raw data underlying the present study are shared as Supplementary Materials. Additional information can be obtained from the author upon reasonable request.

## Supplementary Materials

The following information can be downloaded at: https://www.mdpi.com/article/10.3390/molecules29204788/s1: (1) Selected optimized geometric parameters, NCIPLOT, QTAIM, ESI-3D, NBO and IboView data. (2) The XYZ coordinates (in Å) for pincer ligands and their agostic complexes with Rh(I), both with and without ancillary ligands, in their optimized geometries.
