# Supplementary material for: Electron Density and Molecular Orbital Analyses of the Nature of Bonding in the η3-CCH Agostic Rhodium Complexes Preceding the C–C and C–H Bond Cleavages
Source: Molecules. 2024 Oct 10;29(20):4788. doi: 10.3390/molecules29204788 (PMC11510536; doi:10.3390/molecules29204788)
Supplement: Supplementary file 1 [file molecules-29-04788-s001.zip › dynamic eta3-SI-edited.pdf]

Supporting Information for the paper

Electron Density and Molecular Orbital Analyses of the Nature of Bonding in the η^3-CCH Agostic Rhodium Complexes Preceding the C−C and C−H Bond Cleavages.

By Irena Efremenko

Table S1. Selected optimized geometric parameters of η[3] agostic Rh-$C_{sp^2}C_{sp^3}$H complexes.

	Bond distances, Å			Angles and dihedral angles, deg			
Pincer ligand	Rh-C_{sp2}	Rh-C_{sp3}	Rh-H	∠Rh-C_{ipso}-C_{sp3}	∠Rh-C_{sp3}-H	∠Rh-C_{ipso}-C_{para}	∢Rh-C_{ipso}-C_{sp3}-H
blank							
PCP [a]	2.416	2.218	1.694	64.0	49.1	130.4	7.1
PCN	2.176	2.309	1.913	74.7	55.7	125.4	-0.4
PCO [b]	2.296	2.188	1.711	66.4	51.1	127.1	-2.7
POCOP [c]	2.214	2.239	1.828	70.7	54.6	133.4	2.2
SCS	2.163	2.101	1.664	67.0	52.4	148.9	2.4
MeOH							
PCP	2.322	2.342	1.864	71.6	51.7	132.4	0.4
PCN	2.227	2.265	1.815	71.6	52.7	127.0	-3.2
PCO	2.332	2.197	1.690	65.8	49.7	126.0	-11.3
POCOP	2.162	2.372	2.004	77.7	57.4	132.9	-0.2
SCS	2.263	2.151	1.649	66.2	49.7	137.0	0.8
C_2H_4							
PCP	2.388	2.467	1.939	74.7	49.7	123.7	-1.3
PCN	2.287	2.475	2.023	78.2	53.6	124.7	-4.0
PCO	2.289	2.450	1.980	77.3	52.6	118.0	-7.8
POCOP	2.220	2.487	2.079	80.9	55.9	128.8	-2.4
SCS	2.345	2.518	2.046	78.4	52.8	114.7	19.2
CO							
PCP	2.385	2.468	1.957	74.8	50.6	124.8	3.2
PCN	2.287	2.550	2.126	81.3	55.4	121.0	-3.1
PCO	2.313	2.631	2.210	83.9	55.9	115.3	-0.6
POCOP	2.240	2.505	2.095	81.1	55.9	126.1	0.0
SCS	2.399	2.356	1.861	69.8	50.8	131.3	0.8

[a] Rh-*t*Bu bonding; Rh-C=2.531 Å, Rh-H=1.845 Å; [b] Rh-tBu bonding; Rh-C=2.550 Å, Rh-H=1.892 Å; [c] Rh-*t*Bu bonding; Rh-C=2.641 Å, Rh-H=1.912 Å.

Table S2. QTAIM charges on select atoms and groups in the free pincer ligands and in their η^3 agostic Rh(I) complexes.

Ancillary ligand	Pincer ligand	Rh	P/S [a]	P/N/O/S [b]	C_{ipso}	C_{Me}	H_{Me}	Ancillary ligand		
								O,C [c]	O,C [d]	Total
Uncoordinated pincer ligand	PCP		1.356	1.356	-0.015	0.021	0.042			
	POCOP		1.644	1.637	-0.003	0.030	0.032			
	PCN		1.350	-1.032	-0.012	0.033	0.039			
	PCO		1.347	-1.081	-0.010	0.008	-0.012			
	SCS		-1.001	-1.007	0.017	0.003	0.014			
No ancillary ligand	PCP	0.158	1.575	1.428	-0.030	-0.082	0.006			
	POCOP	0.176	1.847	1.705	-0.048	-0.064	0.008			
	PCN	0.340	1.559	-1.004	-0.080	-0.039	0.007			
	PCO	0.261	1.674	-1.080	-0.043	-0.090	0.014			
	SCS	0.463	-0.704	-0.732	0.011	-0.080	0.057			
MeOH	PCP	0.253	1.487	1.465	-0.090	-0.056	0.033	-1.142		0.070
	POCOP	0.247	1.706	1.724	-0.089	-0.027	0.015	-1.137		0.075
	PCN	0.366	1.510	-0.980	-0.074	-0.078	0.022	-1.140		0.073
	PCO	0.346	1.528	-1.076	-0.038	-0.096	0.029	-1.143		0.098
	SCS	0.478	-0.727	-0.734	0.015	-0.079	0.054	-1.132		0.099
CO	PCP	0.239	1.474	1.462	-0.122	-0.041	0.020	1.036	-1.203	-0.168
	POCOP	0.236	1.719	1.718	-0.132	-0.005	0.015	1.035	-1.195	-0.160
	PCN	0.361	1.558	-0.980	-0.144	-0.006	0.029	1.036	-1.198	-0.136
	PCO	0.369	1.526	-1.084	-0.145	-0.017	0.029	1.053	-1.189	-0.161
	SCS	0.410	-0.660	-0.670	-0.021	-0.056	0.036	1.098	-1.173	-0.075
C_2H_4	PCP	0.286	1.430	1.412	-0.106	-0.032	0.020	-0.087	-0.080	-0.098
	POCOP	0.254	1.677	1.683	-0.125	-0.005	0.023	-0.087	-0.085	-0.077
	PCN	0.388	1.474	-0.979	-0.117	-0.029	0.030	-0.091	-0.084	-0.090
	PCO	0.390	1.515	-1.086	-0.113	-0.030	0.017	-0.092	-0.091	-0.081
	SCS	0.422	-0.718	-0.694	-0.065	-0.020	0.040	-0.080	-0.080	-0.006

[a] Sidearm P atom in each pincer ligand except SCS, and sidearm S atom in SCS.
[b] Second ligating atom from the sidearm of each pincer ligand.
[c] O-atom of MeOH, C atoms of CO and C_2H_4.
[d] O-atom of CO, second C atom of C_2H_4.

Table S3. QTAIM properties of selected Line Critical Points (*lcp*) in η^3 agostic Rh(I) complexes

	LPL	LPL-GBL	ρ	$\nabla^2\rho$	K	V	V_{rep}	Ellipt.	DI	q(A|B)
					**Rh-P/S**					
Rh-PCP	4.262	0.014	0.116	0.099	0.054	-0.133	6.929	0.009	0.939	-0.231
Rh-POCOP	4.243	0.015	0.120	0.100	0.057	-0.139	7.041	0.022	0.963	-0.124
Rh-PCN	4.265	0.017	0.115	0.121	0.053	-0.135	6.396	0.077	1.024	-0.058
Rh-PCO	4.107	0.010	0.137	0.024	0.077	-0.160	7.341	0.031	1.119	-0.268
Rh-SCS	4.403	0.002	0.090	0.205	0.029	-0.108	4.733	0.124	0.936	0.146
Rh-PCP-MeOH	4.398	0.013	0.099	0.141	0.038	-0.112	6.090	0.106	0.896	-0.133
Rh-POCOP-MeOH	4.404	0.019	0.100	0.143	0.039	-0.113	6.021	0.084	0.891	0.033
Rh-PCN-MeOH	4.289	0.013	0.112	0.117	0.050	-0.128	6.537	0.088	1.021	-0.113
Rh-PCO-MeOH	4.241	0.012	0.118	0.084	0.056	-0.133	6.553	0.074	1.094	-0.055
Rh-SCS-MeOH	4.455	0.002	0.084	0.202	0.025	-0.101	4.680	0.171	0.847	0.185
Rh-CO-PCP	4.432	0.008	0.098	0.104	0.038	-0.103	5.980	0.079	0.836	-0.082
Rh-CO-POCOP	4.407	0.010	0.102	0.110	0.041	-0.109	6.099	0.097	0.846	-0.071
Rh-CO-PCN	4.302	0.008	0.112	0.081	0.051	-0.121	6.477	0.092	0.962	-0.132
Rh-CO-PCO	4.231	0.006	0.121	0.047	0.060	-0.131	6.683	0.097	1.033	-0.136
Rh-CO-SCS	4.462	0.009	0.086	0.178	0.027	-0.097	4.723	0.047	0.853	0.112
Rh-C_2H_4-PCP	4.520	0.014	0.089	0.122	0.031	-0.093	5.424	0.045	0.800	-0.123
Rh- C_2H_4-POCOP	4.439	0.016	0.098	0.127	0.037	-0.106	5.872	0.044	0.840	-0.070
Rh- C_2H_4-PCN	4.344	0.014	0.107	0.107	0.045	-0.117	6.155	0.024	0.975	-0.126
Rh- C_2H_4-PCO	4.254	0.011	0.118	0.075	0.056	-0.130	6.533	0.037	1.051	-0.144
Rh- C_2H_4-SCS	4.474	0.003	0.082	0.196	0.024	-0.097	4.564	0.134	0.824	0.118
					**Rh-P/N/O/S**					
Rh-PCP	4.484	0.013	0.092	0.137	0.033	-0.100	5.381	0.169	0.823	0.101
Rh-POCOP	4.487	0.019	0.093	0.138	0.033	-0.101	5.343	0.163	0.824	-0.014
Rh-PCN	4.090	0.006	0.086	0.315	0.020	-0.119	4.578	0.131	0.555	0.180
Rh-PCO	4.281	0.022	0.057	0.273	0.006	-0.079	2.714	0.340	0.357	0.249
Rh-SCS	4.423	0.003	0.088	0.199	0.028	-0.106	4.672	0.110	0.918	0.146
Rh-PCP-MeOH	4.449	0.017	0.095	0.134	0.035	-0.104	5.798	0.099	0.858	-0.031
Rh-POCOP-MeOH	4.389	0.015	0.102	0.138	0.040	-0.115	6.159	0.060	0.887	0.031
Rh-PCN-MeOH	4.225	0.013	0.073	0.271	0.014	-0.096	4.070	0.149	0.496	0.162
Rh-PCO-MeOH	4.278	0.020	0.056	0.274	0.005	-0.079	2.836	0.355	0.343	0.054
Rh-SCS-MeOH	4.471	0.008	0.084	0.194	0.025	-0.098	4.664	0.121	0.849	0.152
Rh-CO-PCP	4.425	0.008	0.099	0.104	0.039	-0.103	6.023	0.086	0.841	-0.082
Rh-CO-POCOP	4.407	0.010	0.102	0.110	0.041	-0.109	6.096	0.097	0.845	-0.065
Rh-CO-PCN	4.171	0.006	0.080	0.270	0.018	-0.102	4.415	0.047	0.513	0.144
Rh-CO-PCO	4.197	0.014	0.063	0.298	0.007	-0.089	3.147	0.126	0.376	0.169
Rh-CO-SCS	4.464	0.009	0.086	0.176	0.027	-0.097	4.724	0.048	0.855	0.098
Rh-C_2H_4-PCP	4.516	0.013	0.090	0.119	0.032	-0.093	5.480	0.056	0.804	-0.010
Rh- C_2H_4-POCOP	4.451	0.015	0.097	0.122	0.037	-0.104	5.839	0.076	0.828	-0.068
Rh- C_2H_4-PCN	4.324	0.016	0.067	0.228	0.012	-0.081	3.674	0.099	0.462	0.138
Rh- C_2H_4-PCO	4.269	0.027	0.057	0.275	0.005	-0.080	2.868	0.193	0.338	0.161
Rh- C_2H_4-SCS	4.448	0.004	0.085	0.198	0.026	-0.101	4.716	0.096	0.830	0.107

	LPL	LPL-GBL	ρ	$\nabla^2\rho$	K	V	V_{rep}	Ellipt.	DI	q(A|B)
**Rh-C_{ipso}**										
Rh-POCOP	4.210	0.026	0.081	0.185	0.023	-0.092	4.658	0.549	0.436	0.143
Rh-PCN	4.124	0.010	0.085	0.175	0.026	-0.096	4.587	0.206	0.480	0.138
Rh-SCS	4.140	0.054	0.095	0.187	0.031	-0.109	5.232	1.031	0.569	0.120
Rh-PCP-MeOH	4.425	0.038	0.061	0.161	0.013	-0.066	3.698	0.469	0.377	0.185
Rh-POCOP-MeOH	4.094	0.009	0.087	0.184	0.028	-0.101	5.264	0.022	0.524	0.182
Rh-PCN-MeOH	4.243	0.033	0.076	0.178	0.021	-0.085	4.287	0.409	0.415	0.130
Rh-CO-PCP	4.537	0.031	0.050	0.156	0.008	-0.054	2.963	0.434	0.274	0.112
Rh-CO-POCOP	4.234	0.002	0.070	0.190	0.017	-0.081	4.141	0.025	0.393	0.128
Rh-CO-PCN	4.324	0.003	0.061	0.174	0.012	-0.068	3.386	0.041	0.330	0.089
Rh-CO-PCO	4.373	0.002	0.057	0.166	0.011	-0.063	2.981	0.092	0.308	0.088
Rh-CO-SCS	4.676	0.142	0.053	0.152	0.008	-0.055	2.941	1.807	0.276	0.055
Rh-C_2H_4-PCP	4.553	0.040	0.052	0.145	0.009	-0.054	3.081	0.218	0.285	0.118
Rh- C_2H_4-POCOP	4.198	0.002	0.076	0.178	0.021	-0.085	4.523	0.195	0.433	0.126
Rh- C_2H_4-PCN	4.330	0.007	0.064	0.161	0.014	-0.069	3.569	0.124	0.349	0.091
Rh- C_2H_4-PCO	4.345	0.019	0.063	0.169	0.014	-0.070	3.316	0.084	0.324	0.095
Rh- C_2H_4-SCS	4.450	0.017	0.055	0.147	0.010	-0.057	3.085	0.142	0.316	0.067
**Rh-H**										
Rh-PCP	3.240	0.043	0.102	0.258	0.043	-0.150	5.764	0.386	0.421	0.202
Rh-POCOP	3.658	0.206	0.080	0.272	0.019	-0.106	4.395	1.665	0.309	0.081
Rh-PCN	3.984	0.369	0.068	0.246	0.012	-0.085	3.454	2.838	0.254	0.073
Rh-PCO	3.289	0.061	0.100	0.273	0.040	-0.147	4.885	0.579	0.415	0.189
Rh-SCS	3.192	0.057	0.116	0.250	0.054	-0.169	5.963	0.843	0.462	0.043
Rh-PCP-MeOH	3.623	0.099	0.073	0.232	0.018	-0.094	4.195	0.491	0.271	0.133
Rh-PCN-MeOH	3.531	0.103	0.081	0.257	0.023	-0.110	4.359	0.655	0.317	0.085
Rh-PCO-MeOH	3.234	0.045	0.104	0.257	0.045	-0.154	5.337	0.333	0.419	0.149
Rh-SCS-MeOH	3.144	0.036	0.115	0.241	0.057	-0.173	6.286	0.324	0.460	0.100
Rh-CO-PCP	3.786	0.084	0.057	0.191	0.010	-0.068	3.231	0.330	0.212	0.086
Rh-CO-POCOP	4.266	0.304	0.046	0.167	0.004	-0.050	2.511	2.190	0.163	0.051
Rh-CO-PCN	4.257	0.236	0.043	0.151	0.004	-0.046	2.204	1.374	0.149	0.050
Rh-CO-PCO	4.587	0.406	0.037	0.129	0.003	-0.037	1.751	5.473	0.128	0.049
Rh-CO-SCS	3.621	0.104	0.066	0.249	0.013	-0.088	3.530	0.430	0.234	0.031
Rh-C_2H_4-PCP	3.739	0.070	0.058	0.195	0.011	-0.070	3.281	0.239	0.217	0.091
Rh- C_2H_4-POCOP	4.208	0.276	0.047	0.174	0.004	-0.052	2.589	1.879	0.160	0.054
Rh- C_2H_4-PCN	3.957	0.130	0.052	0.176	0.007	-0.058	2.675	0.477	0.180	0.050
Rh- C_2H_4-PCO	3.867	0.122	0.054	0.199	0.008	-0.065	2.649	0.810	0.200	0.068
Rh- C_2H_4-SCS	3.998	0.128	0.045	0.174	0.004	-0.052	2.350	0.633	0.175	0.027
**C_{ipso}-C(Me)**										
PCP	2.837	0.000	0.258	-0.672	0.232	-0.297	9.977	0.036	1.016	
POCOP	2.828	0.000	0.258	-0.670	0.234	-0.300	9.686	0.032	1.017	
PCN	2.835	0.000	0.259	-0.676	0.233	-0.298	8.969	0.035	1.016	
PCO	2.835	0.000	0.258	-0.675	0.233	-0.298	8.183	0.033	1.018	
SCS	2.827	0.000	0.260	-0.685	0.239	-0.307	9.616	0.034	1.021	
Rh-PCP	2.855	0.000	0.251	-0.622	0.222	-0.121	12.687	0.068	0.984	
Rh-POCOP	2.900	0.001	0.235	-0.534	0.199	-0.289	11.725	0.088	0.941	
Rh-PCN	2.907	0.001	0.236	-0.544	0.199	-0.264	10.912	0.081	0.948	
Rh-PCO	2.866	0.000	0.248	-0.601	0.217	-0.262	10.887	0.082	0.977	
Rh-SCS	2.871	0.001	0.247	-0.582	0.216	-0.286	12.049	0.114	0.962	

	LPL	LPL-GBL	ρ	$\nabla^2\rho$	K	V	V_{rep}	Ellipt.	DI	q(A|B)
Rh-PCP-MeOH	2.893	0.001	0.237	-0.551	0.203	-0.267	12.395	0.091	0.966	
Rh-POCOP-MeOH	2.918	0.002	0.227	-0.500	0.189	-0.253	11.693	0.091	0.929	
Rh-PCN-MeOH	2.877	0.001	0.243	-0.570	0.210	-0.277	11.779	0.104	0.969	
Rh-PCO-MeOH	2.840	0.000	0.255	-0.637	0.229	-0.298	11.689	0.083	0.992	
Rh-SCS-MeOH	2.823	0.000	0.261	-0.659	0.238	-0.310	13.201	0.096	1.003	
Rh-CO-PCP	2.869	0.001	0.245	-0.592	0.213	-0.284	12.505	0.077	0.992	
Rh-CO-POCOP	2.879	0.002	0.238	-0.559	0.206	-0.279	11.961	0.073	0.967	
Rh-CO-PCN	2.882	0.001	0.241	-0.577	0.208	-0.272	11.300	0.070	0.982	
Rh-CO-PCO	2.877	0.001	0.243	-0.590	0.211	-0.272	10.632	0.060	0.987	
Rh-CO-SCS	2.873	0.002	0.246	-0.601	0.215	-0.275	12.110	0.066	0.984	
Rh-C_2H_4-PCP	2.857	0.001	0.249	-0.615	0.220	-0.279	12.807	0.069	0.994	
Rh- C_2H_4-POCOP	2.881	0.002	0.238	-0.560	0.205	-0.285	12.085	0.069	0.960	
Rh- C_2H_4-PCN	2.878	0.001	0.243	-0.582	0.210	-0.271	11.515	0.072	0.980	
Rh- C_2H_4-PCO	2.860	0.001	0.249	-0.612	0.219	-0.274	11.137	0.074	0.993	
Rh- C_2H_4-SCS	2.845	0.000	0.254	-0.641	0.227	-0.285	12.461	0.068	1.004	
**C(Me)-H(Me)**										
PCP	2.028	0.000	0.283	-1.022	0.300	-0.344	10.045	0.006	0.913	
POCOP	2.029	0.000	0.283	-1.021	0.300	-0.344	9.637	0.008	0.922	
PCN	2.027	0.000	0.283	-1.022	0.300	-0.345	8.883	0.005	0.918	
PCO	2.041	0.000	0.275	-0.962	0.289	-0.338	7.539	0.015	0.944	
SCS	2.028	0.000	0.281	-1.003	0.298	-0.346	9.261	0.013	0.920	
Rh-PCP	2.278	0.006	0.204	-0.507	0.176	-0.226	10.127	0.082	0.652	
Rh-POCOP	2.178	0.002	0.230	-0.669	0.216	-0.265	10.897	0.044	0.741	
Rh-PCN	2.131	0.001	0.244	-0.760	0.238	-0.286	10.552	0.020	0.790	
Rh-PCO	2.272	0.006	0.207	-0.520	0.179	-0.229	8.710	0.083	0.665	
Rh-SCS	2.338	0.009	0.192	-0.436	0.159	-0.208	8.755	0.142	0.601	
Rh-PCP-MeOH	2.132	0.001	0.245	-0.763	0.239	-0.286	12.303	0.020	0.766	
Rh-POCOP-MeOH	2.095	0.000	0.258	-0.850	0.259	-0.306	12.420	0.006	0.817	
Rh-PCN-MeOH	2.173	0.001	0.233	-0.678	0.218	-0.267	10.776	0.041	0.733	
Rh-PCO-MeOH	2.271	0.006	0.208	-0.521	0.180	-0.230	9.275	0.093	0.648	
Rh-SCS-MeOH	2.316	0.010	0.200	-0.472	0.168	-0.217	9.631	0.118	0.610	
Rh-CO-PCP	2.104	0.001	0.254	-0.824	0.253	-0.301	12.454	0.008	0.800	
Rh-CO-POCOP	2.074	0.000	0.265	-0.901	0.272	-0.319	12.490	0.004	0.838	
Rh-CO-PCN	2.068	0.000	0.267	-0.915	0.275	-0.321	11.687	0.007	0.847	
Rh-CO-PCO	2.057	0.000	0.271	-0.942	0.281	-0.327	10.959	0.009	0.867	
Rh-CO-SCS	2.117	0.002	0.250	-0.792	0.246	-0.295	11.581	0.033	0.781	
Rh-C_2H_4-PCP	2.106	0.001	0.253	-0.816	0.252	-0.299	12.517	0.009	0.795	
Rh- C_2H_4-POCOP	2.072	0.000	0.266	-0.907	0.273	-0.319	12.667	0.002	0.837	
Rh- C_2H_4-PCN	2.083	0.000	0.261	-0.872	0.265	-0.312	11.658	0.006	0.826	
Rh- C_2H_4-PCO	2.103	0.001	0.254	-0.826	0.254	-0.301	10.768	0.011	0.812	
Rh- C_2H_4-SCS	2.091	0.000	0.259	-0.865	0.261	-0.306	11.879	0.016	0.825	
**Rh-O(MeOH)**										
Rh-PCP-MeOH	4.095	0.004	0.069	0.357	0.009	-0.108	4.062	0.186	0.489	0.091
Rh-POCOP-MeOH	4.112	0.002	0.067	0.349	0.008	-0.103	3.816	0.195	0.466	0.098
Rh-PCN-MeOH	4.121	0.005	0.066	0.349	0.008	-0.102	3.571	0.265	0.446	0.075
Rh-PCO-MeOH	4.047	0.021	0.075	0.380	0.011	-0.117	3.827	0.082	0.490	0.054
Rh-SCS-MeOH	3.979	0.005	0.080	0.414	0.013	-0.129	4.359	0.332	0.502	0.035

	LPL	LPL-GBL	ρ	$\nabla^2\rho$	K	V	V_{rep}	Ellipt.	DI	q(A|B)
					**Rh-CO**					
Rh-CO-PCP	3.397	0.000	0.195	0.441	0.129	-0.367	11.947	0.016	1.481	0.199
Rh-CO-POCOP	3.440	0.000	0.184	0.457	0.116	-0.344	10.985	0.026	1.419	0.187
Rh-CO-PCN	3.413	0.000	0.190	0.472	0.123	-0.362	10.768	0.024	1.472	0.199
Rh-CO-PCO	3.409	0.001	0.192	0.465	0.125	-0.364	10.219	0.027	1.493	0.183
Rh-CO-SCS	3.426	0.000	0.189	0.450	0.121	-0.353	10.499	0.128	1.406	0.106
					**(Rh)C-O**					
CO	2.123	0.000	0.511	0.882	0.954	-2.129	5.075	0.000	1.751	
Rh-CO-PCP	2.166	0.000	0.483	0.606	0.902	-1.955	23.916	0.006	1.466	
Rh-CO-POCOP	2.160	0.000	0.487	0.643	0.910	-1.980	23.224	0.006	1.494	
Rh-CO-PCN	2.163	0.000	0.485	0.624	0.904	-1.964	21.790	0.003	1.482	
Rh-CO-PCO	2.160	0.000	0.487	0.650	0.909	-1.980	20.432	0.002	1.493	
Rh-CO-SCS	2.151	0.000	0.492	0.720	0.920	-2.020	21.452	0.002	1.523	
					**Rh-C_1(C_2H_4)**					
Rh-C_2H_4-PCP	3.953	0.022	0.113	0.169	0.045	-0.132	6.595	0.538	0.787	0.108
Rh- C_2H_4-POCOP	4.028	0.033	0.105	0.178	0.038	-0.121	5.956	0.636	0.731	0.105
Rh- C_2H_4-PCN	3.948	0.021	0.114	0.170	0.046	-0.133	6.107	0.468	0.782	0.114
Rh- C_2H_4-PCO	3.956	0.023	0.113	0.190	0.044	-0.136	5.749	0.706	0.782	0.105
Rh- C_2H_4-SCS	3.987	0.035	0.111	0.185	0.043	-0.131	5.782	0.807	0.767	0.043
					**Rh-C_2(C_2H_4)**					
Rh-C_2H_4-PCP	3.981	0.022	0.110	0.186	0.042	-0.130	6.451	0.723	0.770	0.100
Rh- C_2H_4-POCOP	4.041	0.033	0.103	0.187	0.037	-0.120	5.909	0.762	0.730	0.100
Rh- C_2H_4-PCN	3.994	0.024	0.108	0.194	0.040	-0.129	5.849	0.809	0.767	0.103
Rh- C_2H_4-PCO	3.923	0.019	0.117	0.166	0.049	-0.139	6.010	0.509	0.797	0.098
Rh- C_2H_4-SCS	3.934	0.022	0.116	0.160	0.048	-0.136	6.124	0.461	0.791	0.052
					**C_1-C_2(C_2H_4)**					
C_2H_4	2.500	0.000	0.357	-1.106	0.426	-0.575	3.344	0.328	1.904	
Rh-C_2H_4-PCP	2.662	0.003	0.303	-0.839	0.315	-0.420	15.056	0.216	1.251	
Rh- C_2H_4-POCOP	2.646	0.003	0.309	-0.866	0.325	-0.434	14.799	0.223	1.288	
Rh- C_2H_4-PCN	2.658	0.003	0.305	-0.845	0.318	-0.424	13.748	0.220	1.264	
Rh- C_2H_4-PCO	2.669	0.003	0.301	-0.824	0.311	-0.416	12.794	0.224	1.252	
Rh- C_2H_4-SCS	2.665	0.003	0.303	-0.838	0.314	-0.419	13.162	0.216	1.258	

Notations: LPL = Line path length; GBP = Geometric bond length; ρ = Electron density; $\nabla^2\rho$ = Laplacian of electron density = Trace of Hessian of ρ; K = Hamiltonian form of kinetic energy density; V = Virial field = Potential energy density = Trace of stress tensor; V_{rep} = V - V_{en} = Repulsive contribution to virial field V, where V_{en} = Electron-nuclear attractive contribution to virial field V; Ellipt. = Bond ellipticity; DI = Electron Delocalization Index, Average Number of Electrons Delocalized (Shared) Between Atoms A and B; q(A|B) = Contribution of bond between atom A and atom B to q(A), A=Rh. All values are in atomic units.

Table S4. QTAIM properties of selected Ring Critical Points (*rcp*) in η^3 agostic Rh complexes

Ligands	Atoms	ρ	$\nabla^2\rho$	K	Atoms	ρ	$\nabla^2\rho$	K	Atoms	ρ	$\nabla^2\rho$	K
	rcp$_2$				*rcp*$_3$				*rcp*$_4$			
PCP	7	0.023	0.081	-0.001	7	0.021	0.074	-0.001				
POCOP	5	0.030	0.110	0.000	5	0.027	0.099	0.000	4	0.074	0.229	0.015
PCN	8	0.006	0.025	-0.001	5	0.028	0.111	0.000	4	0.067	0.224	0.012
PCO	7	0.026	0.092	0.000	7	0.021	0.095	-0.002				
SCS	5	0.022	0.075	0.000	5	0.021	0.074	-0.001	4	0.092	0.271	0.024
PCP-MeOH	5	0.023	0.080	0.000	5	0.024	0.081	0.000	4	0.058	0.196	0.009
POCOP-MeOH	5	0.030	0.107	0.000	5	0.030	0.108	0.000				
PCN-MeOH	5	0.026	0.092	0.000	5	0.025	0.101	-0.001	4	0.070	0.229	0.014
PCO-MeOH	7	0.024	0.087	-0.001	7	0.021	0.095	-0.002				
SCS-MeOH	7	0.020	0.069	-0.001	7	0.020	0.069	-0.001				
PCP-C_2H_4	5	0.022	0.074	0.000	5	0.022	0.072	0.000	4	0.047	0.174	0.005
POCOP- C_2H_4	5	0.029	0.100	0.000	5	0.029	0.097	0.000	4	0.046	0.183	0.004
PCN- C_2H_4	5	0.025	0.084	0.000	5	0.024	0.092	0.000	4	0.047	0.189	0.004
PCO- C_2H_4	5	0.026	0.087	0.000	5	0.023	0.099	-0.001	4	0.050	0.194	0.005
SCS- C_2H_4	5	0.023	0.071	0.000	5	0.021	0.066	0.000	4	0.043	0.177	0.003
PCP-CO	5	0.023	0.073	0.000	5	0.023	0.074	0.000	4	0.047	0.170	0.005
POCOP-CO	5	0.029	0.096	0.000	5	0.029	0.096	0.000	4	0.045	0.172	0.004
PCN-CO	5	0.026	0.082	0.000	5	0.026	0.095	0.000	4	0.042	0.159	0.003
PCO-CO	5	0.026	0.083	0.000	5	0.024	0.098	-0.001	4	0.037	0.132	0.002
SCS-CO	5	0.019	0.062	-0.001	5	0.019	0.062	-0.001	4	0.053	0.179	0.007

Notations: ρ = Electron density; $\nabla^2\rho$ = Laplacian of electron density = Trace of Hessian of ρ; K = Hamiltonian form of kinetic energy density. All values are in atomic units.

Table S5. Selected distances between *rcp's* and the nearby *lcp's* (Å) in η^3 agostic Rh(I) complexes.

	distance, Å	No ancillary ligand	MeOH	C_2H_4	CO
Ph-PCP	rcp_2-lcp_{Rh-C}	-	1.131	1.127	1.110
	rcp_2-lcp_{Rh-P}	1.273	1.216	1.186	1.191
	rcp_3-lcp_{Rh-C}	-	1.131	1.122	1.106
	rcp_3-lcp_{Rh-P}	1.230	1.216	1.181	1.185
	rcp_4-lcp_{Rh-C}	-	0.430	0.522	0.486
	rcp_4-lcp_{Rh-H}	-	0.814	0.684	0.713
Rh-POCOP	rcp_2-lcp_{Rh-C}	1.144	1.145	1.131	1.117
	rcp_2-lcp_{Rh-P}	1.117	1.113	1.111	1.103
	rcp_3-lcp_{Rh-C}	1.114	1.138	1.131	1.117
	rcp_3-lcp_{Rh-P}	1.166	1.122	1.098	1.102
	rcp_4-lcp_{Rh-C}	0.505	-	0.936	0.931
	rcp_4-lcp_{Rh-H}	0.727	-	0.217	0.212
Rh-PCN	rcp_2-lcp_{Rh-C}	1.163	1.152	1.139	1.128
	rcp_2-lcp_{Rh-P}	1.200	1.203	1.197	1.180
	rcp_3-lcp_{Rh-C}	1.102	1.098	1.091	1.066
	rcp_3-lcp_{Rh-N}	1.008	0.998	0.968	0.986
	rcp_4-lcp_{Rh-C}	0.807	0.507	0.760	0.873
	rcp_4-lcp_{Rh-H}	0.354	0.772	0.478	0.292
Rh-PCO	rcp_2-lcp_{Rh-C}	-	-	1.152	1.122
	rcp_2-lcp_{Rh-P}	1.264	1.257	1.199	1.183
	rcp_3-lcp_{Rh-C}	-	-	1.080	1.047
	rcp_3-lcp_{Rh-O}	0.917	0.930	0.871	0.889
	rcp_4-lcp_{Rh-C}	-	-	0.734	0.981
	rcp_4-lcp_{Rh-H}	-	-	0.459	0.098
Rh-SCS	rcp_2-lcp_{Rh-C}	1.219	-	1.204	1.155
	rcp_2-lcp_{Rh-S}	1.324	1.323	1.251	1.332
	rcp_3-lcp_{Rh-C}	1.229	-	1.174	1.155
	rcp_3-lcp_{Rh-S}	1.335	1.330	1.280	1.328
	rcp_4-lcp_{Rh-C}	0.310	-	0.749	0.266
	rcp_4-lcp_{Rh-H}	0.817	-	0.414	0.892

Table S6. Electron Sharing Indexes in QTAIM 3D Molecular Space Partition (ESI-3D) for η^3 agostic Rh(I) complexes.

**Ligands**	**4c-ESI**	**3c-ESI**			**2c-ESI (DI)**		
	**Rh-C_{ipso}-C_{Me}-H**	**Rh-C_{ipso}-C_{Me}**	**Rh-C_{Me}-H**	**Rh-C_{ipso}-H**	**Rh-C_{ipso}**	**Rh-C_{Me}**	**Rh-H**
PCP	-0.007	0.054	0.105	-0.004	0.266	0.434	0.421
POCOP	-0.008	0.061	0.092	-0.004	0.436	0.385	0.309
PCN	-0.008	0.061	0.074	-0.002	0.480	0.317	0.254
PCO	-0.009	0.060	0.103	-0.004	0.337	0.447	0.415
SCS	-0.015	0.075	0.112	-0.004	0.569	0.547	0.462
PCP-MeOH	-0.007	0.058	0.068	-0.001	0.377	0.304	0.271
POCOP-MeOH	-0.005	0.057	0.059	-0.001	0.524	0.273	0.209
PCN-MeOH	-0.009	0.063	0.074	-0.002	0.415	0.354	0.317
PCO-MeOH	-0.009	0.058	0.100	-0.003	0.306	0.439	0.419
SCS-MeOH	-0.014	0.069	0.100	-0.003	0.454	0.476	0.460
PCP-C_2H_4	-0.002	0.043	0.060	0.001	0.285	0.206	0.217
POCOP-C_2H_4	-0.003	0.045	0.050	0.001	0.433	0.188	0.160
PCN-C_2H_4	-0.003	0.043	0.052	0.001	0.349	0.195	0.180
PCO-C_2H_4	-0.003	0.044	0.057	0.001	0.324	0.198	0.200
SCS-C_2H_4	-0.003	0.039	0.045	0.001	0.316	0.165	0.175
PCP-CO	-0.002	0.042	0.058	0.000	0.274	0.202	0.212
POCOP-CO	-0.002	0.044	0.049	0.001	0.393	0.177	0.163
PCN-CO	-0.002	0.039	0.001	0.043	0.330	0.159	0.149
PCO-CO	0.000	0.033	0.037	0.001	0.308	0.130	0.128
SCS-CO	-0.003	0.042	0.075	-0.002	0.276	0.241	0.234

Table S7. NPA charges on selected atoms and groups in the free pincer ligands and in their η^3 agostic Rh(I) complexes.

Ancillary ligand	Pincer ligand	Rh	P/S [a]	P/N/O/S [b]	C_{ipso}	C_{Me}	H_{Me}	Ancillary ligand		
								O,C [c]	O,C [d]	Total
Uncoordinated pincer ligand	PCP		1.224	1.224	0.014	-1.009	0.334			
	POCOP		1.510	1.509	-0.209	-0.995	0.338			
	PCN		1.224	-0.366	0.036	-1.009	0.324			
	PCO		1.222	-0.478	0.050	-1.013	0.333			
	SCS		-0.619	-0.641	0.119	-1.036	0.324			
No ancillary ligand	PCP	-0.091	1.206	1.052	-0.022	-0.622	0.240			
	POCOP	-0.047	1.510	1.347	-0.181	-0.647	0.228			
	PCN	0.267	1.156	-0.391	-0.057	-0.659	0.215			
	PCO	0.100	1.369	-0.530	-0.014	-0.632	0.232			
	SCS	0.339	-0.437	-0.428	0.038	-0.631	0.268			
MeOH	PCP	0.037	1.088	1.074	-0.068	-0.662	0.233	-0.679		0.140
	POCOP	-0.022	1.380	1.414	-0.225	-0.648	0.226	-0.679		0.139
	PCN	0.187	1.212	-0.410	-0.045	-0.662	0.226	-0.693		0.125
	PCO	0.157	1.270	-0.525	-0.006	-0.636	0.246	-0.683		0.150
	SCS	0.211	-0.468	-0.446	0.057	-0.634	0.283	-0.666		0.167
CO	PCP	-0.193	1.157	1.155	-0.121	-0.664	0.234	0.617	-0.483	0.134
	POCOP	-0.246	1.465	1.465	-0.275	-0.648	0.229	0.607	-0.464	0.143
	PCN	-0.033	1.274	-0.405	-0.135	-0.665	0.239	0.608	-0.467	0.140
	PCO	-0.003	1.325	-0.518	-0.126	-0.662	0.238	0.613	-0.452	0.161
	SCS	-0.070	-0.406	-0.399	0.024	-0.674	0.264	0.674	-0.430	0.244
C_2H_4 [e]	PCP	0.044	1.142	1.140	-0.096	-0.652	0.237	-0.426	-0.451	-0.044
	POCOP	-0.043	1.461	1.461	-0.250	-0.646	0.237	-0.434	-0.441	-0.018
	PCN	0.210	1.254	-0.408	-0.101	-0.662	0.240	-0.433	-0.456	-0.052
	PCO	0.205	1.340	-0.516	-0.086	-0.654	0.234	-0.452	-0.441	-0.040
	SCS									

[a] Sidearm P atom in each pincer ligand except SCS, and sidearm S atom in SCS.
[b] Second ligating atom from the sidearm of each pincer ligand.
[c] O-atom of MeOH, C atoms of CO and C_2H_4.
[d] O-atom of CO, second C atom of C_2H_4.
[e] Lewis structures with independent C_2H_4 unit are shown.

Table S8. The calculated Wiberg bond indexes in the NAO basis for selected bonds in η^3 agostic Rh(I) complexes.

	Rh-P/S	Rh-P/ N/O/S	Rh-C_{sp2}	Rh-C_{sp3}	C_{sp2}-C_{sp3}	C_{sp3}-H	Rh-H	Rh-L[a]	C-O, C-C[b]
PCP					1.035	0.852			
POCOP					1.033	0.857			
PCN					1.037	0.853			
PCO					1.036	0.854			
SCS					1.048	0.910			
CO									2.159
C_2H_4									2.045
Rh-PCP	0.542	0.414	0.086	0.086	1.022	0.687	0.170		
Rh-POCOP	0.673	0.493	0.255	0.172	0.944	0.704	0.150		
Rh-PCN	0.856	0.258	0.504	0.211	0.972	0.740	0.142		
Rh-PCO	0.971	0.144	0.385	0.128	1.011	0.605	0.269		
Rh-SCS	0.498	0.488	0.343	0.324	1.011	0.630	0.220		
Rh-PCP-MeOH	0.587	0.558	0.371	0.176	0.979	0.734	0.134	0.243	
Rh-POCOP-MeOH	0.488	0.498	0.118	0.254	0.948	0.863	0.040	0.232	
Rh-PCN-MeOH	0.850	0.239	0.445	0.188	0.987	0.683	0.189	0.243	
Rh-PCO-MeOH	0.942	0.157	0.369	0.116	1.027	0.581	0.300	0.273	
Rh-SCS-MeOH	0.740	0.729	0.633	0.263	1.017	0.495	0.401	0.386	
Rh-CO-PCP	0.519	0.523	0.150	0.093	1.012	0.787	0.080	1.475	1.832
Rh-CO-POCOP	0.527	0.527	0.197	0.148	0.980	0.825	0.045	1.018	1.866
Rh-CO-PCN	0.781	0.244	0.227	0.120	1.001	0.821	0.051	1.543	1.815
Rh-CO-PCO	0.881	0.167	0.212	0.114	1.007	0.834	0.043	1.578	1.820
Rh-CO-SCS	0.769	0.775	0.307	0.107	0.980	0.712	0.144	1.648	1.868
Rh-C_2H_4-PCP [c]	0.432	0.432	0.119	0.065	1.016	0.835	0.046	0.513	1.385
Rh- C_2H_4-POCOP [c]	0.534	0.515	0.294	0.203	0.969	0.814	0.047	0.615	1.204
Rh- C_2H_4-PCN [c]	0.806	0.234	0.315	0.156	0.997	0.790	0.070	0.659	1.179
Rh- C_2H_4-PCO [c]	0.902	0.166	0.257	0.136	1.013	0.775	0.091	1.168	1.168
Rh-C_2H_4-SCS									

[a] L stands for an ancillary ligand with L=O for MeOH and L=C for CO and C_2H_4; [b] C-O bond of CO and C-C bond of ethylene ligands. [c] Lewis structures with independent C_2H_4 unit are shown.

Table S9. Second order perturbation theory analysis in NBO basis of dative interactions in η^3 agostic Rh(I) complexes. Interactions weaker than 0.50 kcal/mol are not accounted for.

Ancillary ligand		**PCP**	**POCOP**	**PCN**	**PCO**	**SCS**
No ancillary ligand	C-C → Rh	4.59	10.01	8.53	6.58	16.13
	C-H → Rh	**86.69**	45.63	18.01	**84.54**	**130.88**
	Rh → C-C	-	3.39	3.03	1.06	5.18
	Rh → C-H	4.10	10.19	7.46	19.45	23.78
	Rh → C_{ipso}	1.12	1.40	0.56	11.33	2.53
	Rh → C_{Me}	3.29	3.37	n	9.03	0.63
	Rh → H	4.82	9.08	2.15	2.52	0.82
MeOH	C-C → Rh	7.87	10.45	9.53	7.33	9.06
	C-H → Rh	6.92	15.69	34.29	**99.83**	**143.77**
	Rh → C-C	-	2.59	-	0.55	1.24
	Rh → C-H	4.44	6.10	8.02	22.52	26.58
	Rh → C_{ipso}	3.56	7.52	9.15	10.45	42.14
	Rh → C_{Me}	0.9	2.20	1.82	13.24	12.80
	Rh → H	3.87	5.77	6.73	7.14	12.11
	C_{ipso} → Rh	-	**116.85**	-	-	-
CO	C-C → Rh	-	-	-	-	-
	C-H → Rh	3.43	2.13	1.10	0.62	23.99
	Rh → C-C	0.63	0.99	1.22	-	0.91
	Rh → C-H	6.00	3.45	3.37	2.09	9.35
	Rh → C_{ipso}	36.53	38.53	49.28	**50.20**	**53.00**
	Rh → C_{Me}	3.69	4.97	2.21	-	15.30
	Rh → H	9.95	9.11	8.70	6.23	17.83
	C-C → Rh-C(CO)*	7.29	10.69	7.92	6.63	7.00
	C-H → Rh-C(CO)*	12.02	8.10	5.21	4.23	41.68
C_2H_4 [a]	C-C → Rh	3.51	6.14	5.28	4.91	-
	C-H → Rh	18.21	12.98	9.06	2.42	
	Rh → C-C	6.22	1.05	1.30	1.23	-
	Rh → C-H	0.54	2.27	3.96	4.36	
	Rh → C_{ipso}	22.1	20.01	24.98	27.55	
	Rh → C_{Me}	2.93	1.80	2.22	4.27	
	Rh → H	6.34	1.47	3.52	4.94	
	$\pi_{C-C}(C_2H_4)$ → Rh	**217.05**	**77.53**	**74.43**	**85.63**	
	Rh → $\pi_{C-C}(C_2H_4)$	**79.13**	**67.20**	**80.26**	**82.43**	

[a] Lewis structures with independent C_2H_4 unit are shown.

Table S9. IBO charges on select atoms and groups in the free pincer ligands and in their η^3 agostic Rh(I) complexes.

Ancillary ligand	Pincer ligand	Rh	P/S [a]	P/N/O/S [b]	C_{ipso}	C_{Me}	H_{Me}	Ancillary ligand		
								O,C [c]	O,C [d]	Total
No ancillary ligand	PCP	0.143	0.707	0.606	-0.019	-0.389	0.109			
	POCOP	0.173	0.964	0.851	-0.107	-0.409	0.117			
	PCN	0.284	0.723	-0.214	-0.033	-0.412	0.121			
	PCO	0.215	0.840	-0.399	-0.011	-0.403	0.111			
	SCS	0.394	-0.483	-0.474	0.030	-0.387	0.143			
MeOH	PCP	0.179	0.636	0.630	-0.058	-0.422	0.127	-0.483		0.154
	POCOP	0.163	0.868	0.892	-0.142	-0.417	0.127	-0.494		0.156
	PCN	0.255	0.722	-0.230	-0.032	-0.423	0.116	-0.497		0.149
	PCO	0.247	0.763	-0.397	-0.005	-0.405	0.119	-0.501		0.172
	SCS	0.320	-0.499	-0.494	0.044	-0.401	0.143	-0.502		0.163
CO	PCP	0.245	0.663	0.664	-0.090	-0.431	0.128	0.144	-0.268	-0.123
	POCOP	0.230	0.906	0.906	-0.181	-0.424	0.134	0.136	-0.253	-0.117
	PCN	0.315	0.741	-0.226	-0.097	-0.436	0.143	0.142	-0.258	-0.116
	PCO	0.297	0.796	-0.389	-0.092	-0.438	0.148	0.166	-0.247	-0.081
	SCS	0.269	-0.453	-0.453	0.021	-0.436	0.157	0.212	-0.226	-0.014
C_2H_4	PCP	0.326	0.636	0.636	-0.071	-0.420	0.129	-0.339	-0.360	-0.118
	POCOP	0.291	0.881	0.884	-0.165	-0.421	0.139	-0.348	-0.353	-0.097
	PCN	0.400	0.715	-0.233	-0.074	-0.433	0.141	-0.344	-0.371	-0.122
	PCO	0.400	0.777	-0.389	-0.062	-0.428	0.132	-0.370	-0.363	-0.118
	SCS	0.352	-0.467	-0.460	-0.014	-0.428	0.152	-0.345	-0.349	-0.035

[a] Sidearm P atom in each pincer ligand except SCS, and sidearm S atom in SCS.
[b] Second ligating atom from the sidearm of each pincer ligand.
[c] O-atom of MeOH, C atoms of CO and C_2H_4.
[d] O-atom of CO, second C atom of C_2H_4.
[e] POCOP stands for POCOP-*t*Bu.

Table S10. The calculated Wiberg bond indexes in the IBO basis for selected bonds in η^3 agostic Rh(I) complexes.

	Rh-P/S	Rh-P/ N/O/S	Rh-C_{sp2}	Rh-C_{sp3}	C_{sp2}-C_{sp3}	C_{sp3}-H	Rh-H	Rh-L[a]	C-O, C-C[b]
PCP					1.035	0.927			
POCOP					1.032	0.933			
PCN					1.035	0.931			
PCO					1.034	0.946			
SCS					1.040	0.940			
CO									2.411
C_2H_4									2.033
Rh-PCP	0.598	0.466	0.109	0.273	1.012	0.689	0.218		
Rh-POCOP	0.632	0.476	0.204	0.229	0.960	0.784	0.131		
Rh-PCN	0.703	0.289	0.265	0.174	0.971	0.835	0.101		
Rh-PCO	0.855	0.154	0.153	0.277	1.004	0.697	0.209		
Rh-SCS	0.509	0.495	0.379	0.354	0.999	0.632	0.269		
Rh-PCP-MeOH	0.543	0.521	0.207	0.158	0.982	0.821	0.108	0.253	
Rh-POCOP-MeOH	0.542	0.553	0.284	0.149	0.940	0.866	0.070	0.240	
Rh-PCN-MeOH	0.717	0.260	0.218	0.203	0.986	0.779	0.144	0.234	
Rh-PCO-MeOH	0.805	0.159	0.136	0.280	1.017	0.679	0.224	0.254	
Rh-SCS-MeOH	0.449	0.453	0.293	0.304	1.034	0.630	0.279	0.270	
Rh-CO-PCP	0.493	0.497	0.112	0.082	1.009	0.855	0.074	1.123	2.112
Rh-CO-POCOP	0.506	0.506	0.171	0.073	0.982	0.889	0.048	1.057	2.145
Rh-CO-PCN	0.654	0.260	0.145	0.061	1.001	0.892	0.046	1.124	2.138
Rh-CO-PCO	0.750	0.163	0.135	0.047	1.007	0.904	0.037	1.140	2.157
Rh-CO-SCS	0.446	0.447	0.129	0.120	1.011	0.810	0.106	1.079	2.207
Rh-C_2H_4-PCP	0.482	0.484	0.132	0.089	1.012	0.848	0.079	0.582	1.355
Rh- C_2H_4-POCOP	0.511	0.500	0.216	0.081	0.976	0.888	0.047	0.520	1.400
Rh- C_2H_4-PCN	0.664	0.240	0.174	0.081	0.999	0.872	0.057	0.575	1.375
Rh- C_2H_4-PCO	0.763	0.150	0.150	0.087	1.010	0.854	0.075	0.564	1.358
Rh-C_2H_4-SCS	0.427	0.432	0.174	0.068	1.023	0.859	0.075	0.578	1.364

[a] L stands for an ancillary ligand with L=O for MeOH and L=C for CO and C_2H_4; [b] C-O bond of CO and C-C bond of ethylene ligands.

Table S11. Intrinsic bond orbitals (IBO) responsible for σ donation (1st and 2nd rows) and π backdonation (3rd row) along the reaction pathways for C-C and C-H bond cleavage in Rh-PCN system. Numbers below each orbital indicate the primary orbital composition.

P_{CC}	TS_{CC}	η^3	TS_{CH}	P_{CH}
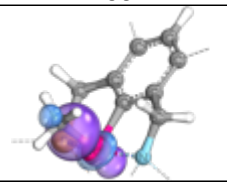	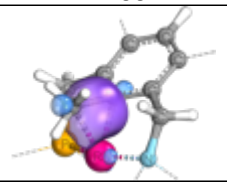	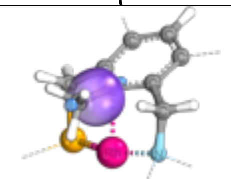	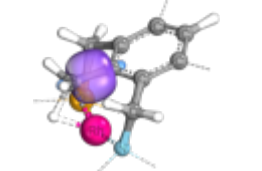	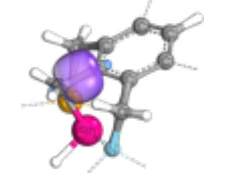
C_i 1.04; Rh 0.94	C_{Me} 0.89; C_i 0.87; Rh 0.22	C_{Me} 0.98; C_i 0.96; Rh 0.05	C_{Me} 1.01; C_i 0.95; Rh 0.02	C_{Me} 1.01; C_i 0.95; Rh 0.02
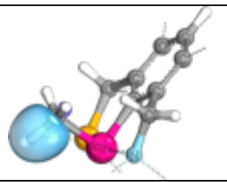	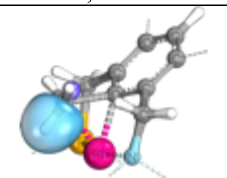	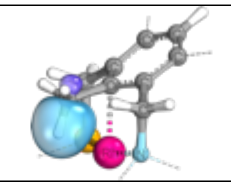	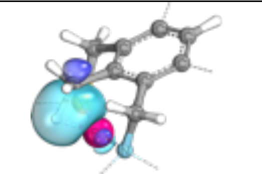	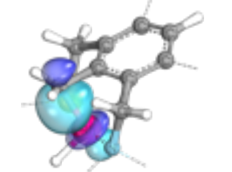
C_{Me} 1.15; H 0.83	C_{Me} 1.12; H 0.82; Rh 0.04	C_{Me} 1.06; H 0.83; Rh 0.08	C_{Me} 0.91; H 0.76; Rh 0.28; C_i 0.03	C_{Me} 0.99; Rh 0.94; C_i 0.03
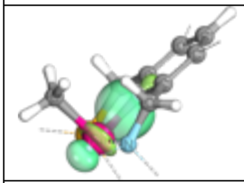	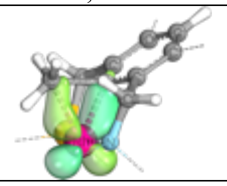	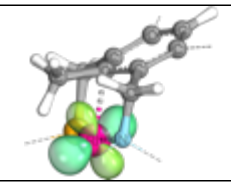	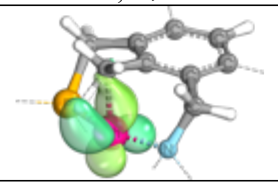	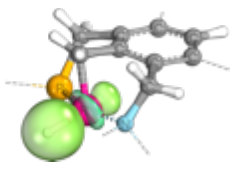
C_i 1.05; Rh 0.92	Rh 1.65; C_i 0.19; C_{Me} 0.1	Rh 1.96	Rh 1.75; H 0.13; C_{Me} 0.08	H 1.05; Rh 0.92

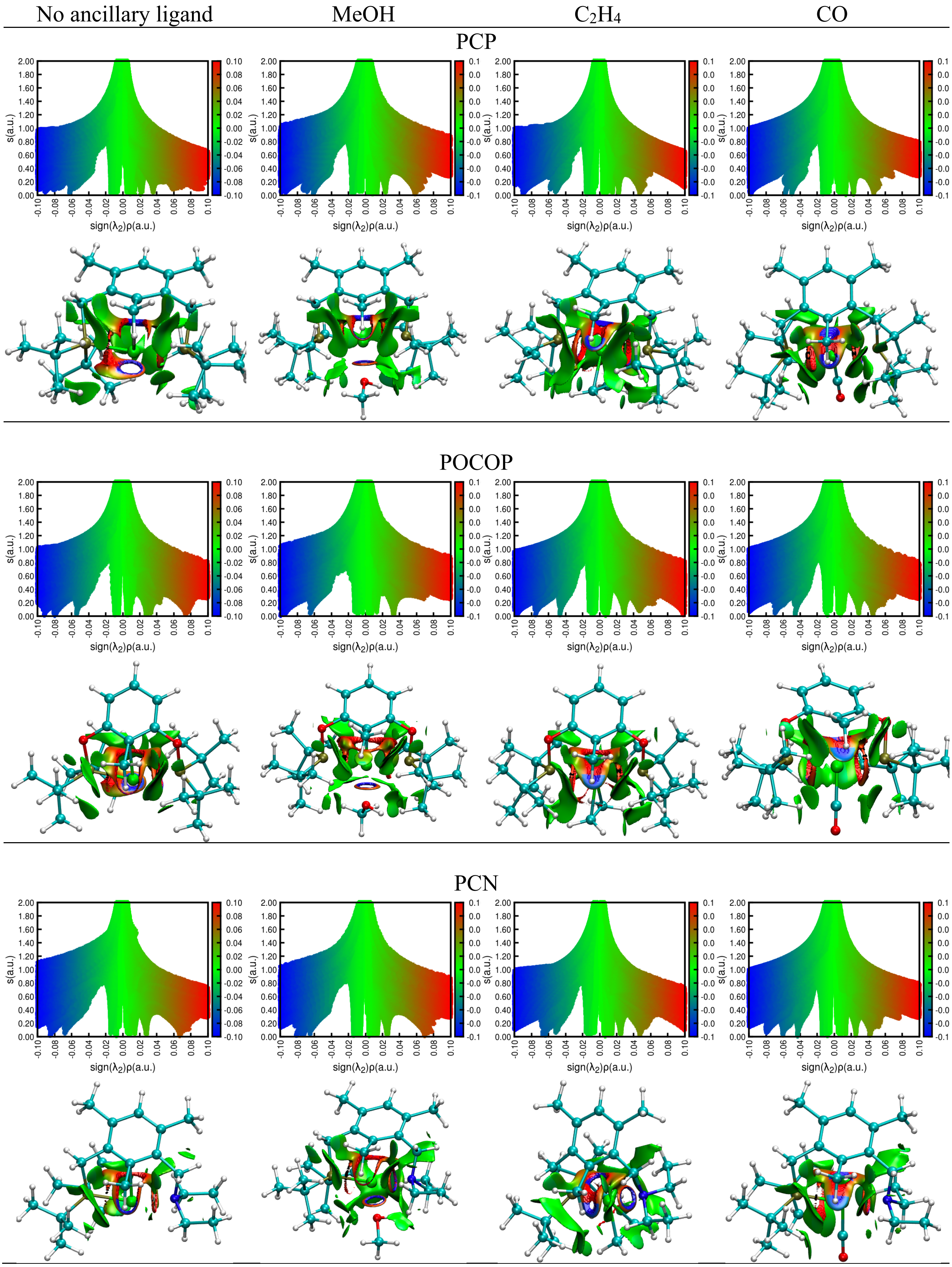
No ancillary ligand
MeOH
C2H4
CO
PCP
POCOP
PCN
s(a.u.)
sign(λ2)ρ(a.u.)

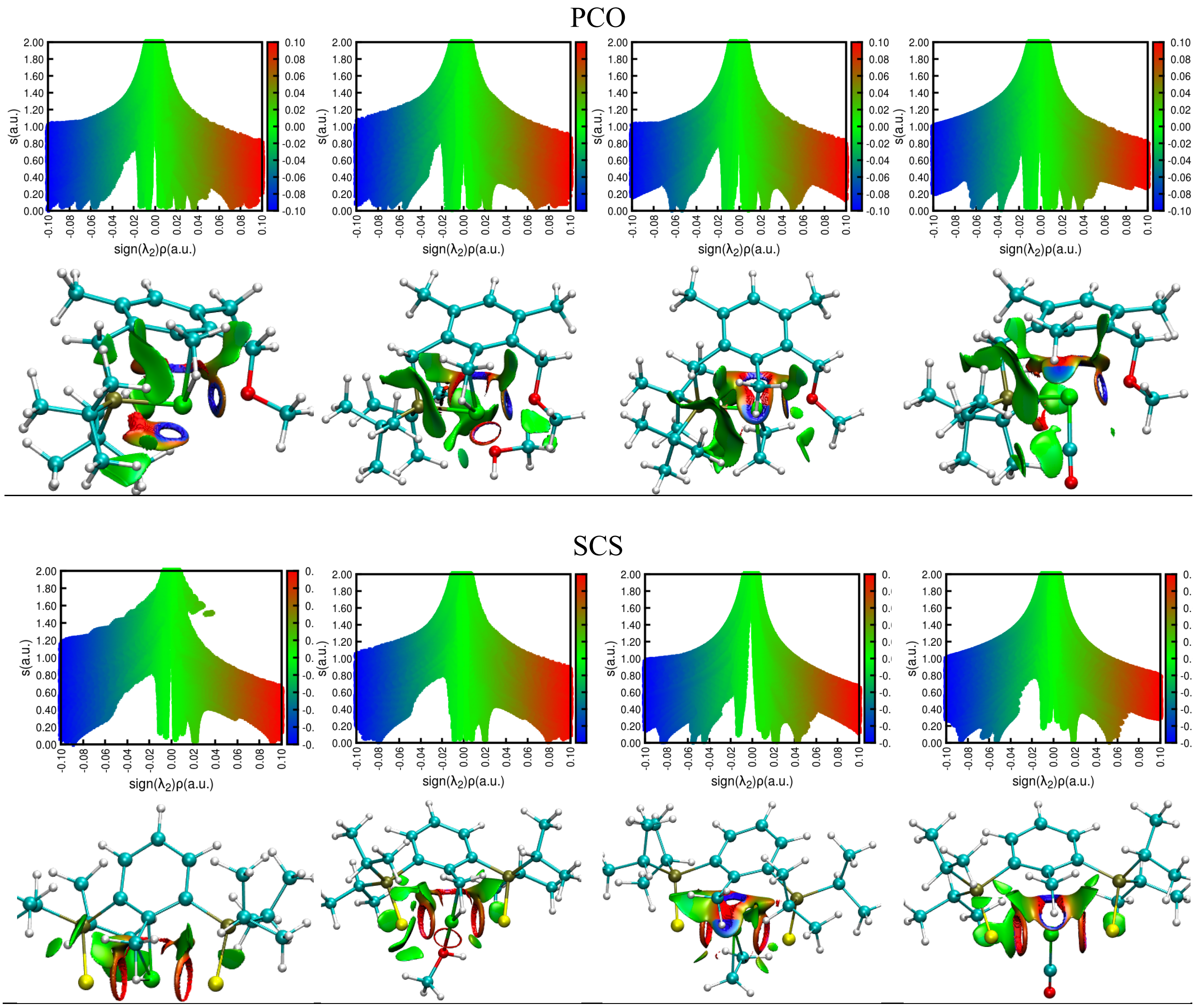

Figure S1. Two-dimensional NCI plots of the reduced density gradient versus the electron density multiplied by the sign of the second Hessian eigenvalue (upper rows) and gradient isosurfaces (s = 0.5 with a ρ(r) = 0.10 cutoff) (lower rows) for η^3 agostic Rh(I) complexes in absence and in presence of ancillary ligands. The surfaces are colored on a blue-green-red scale according to values of sign(λ_2)ρ, ranging from -0.1 to +0.1 au. Blue indicates strong attractive interactions, and red indicates strong repulsive overlap due to the contribution of two atoms to the electron density in the same region. The electron density was analyzed within the radius of 3.0 Å around Rh atom.

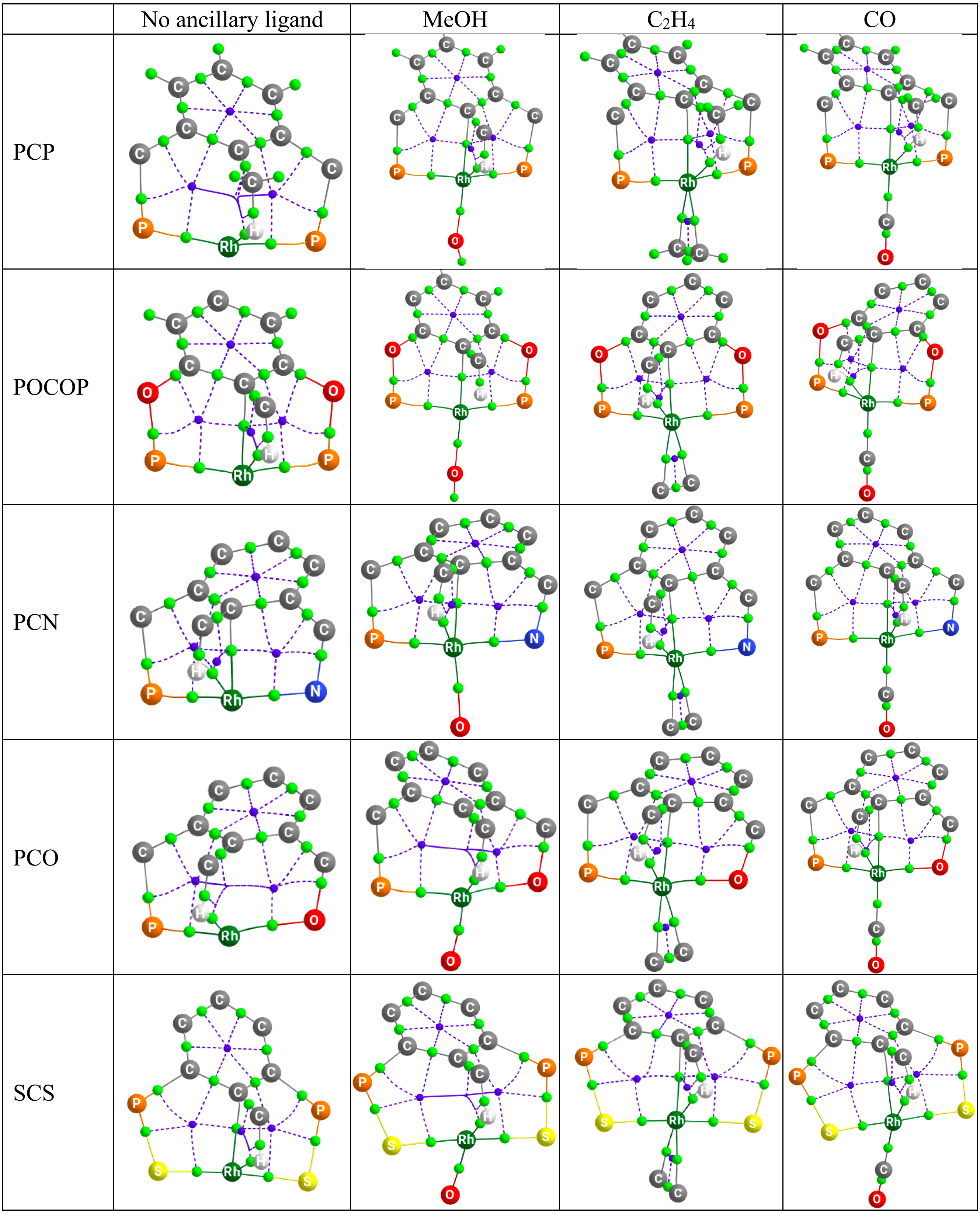

Figure S2. Molecular graphs of η^3 agostic Rh(I) complexes showing line paths (solid atom-colored lines) with line critical points (*lcp*, small green spheres) and ring paths (dashed blue lines) with ring critical points (*rcp*, small blue spheres). Hydrogen atoms and alkyl groups not involved into the agostic bonding are omitted for clarity.

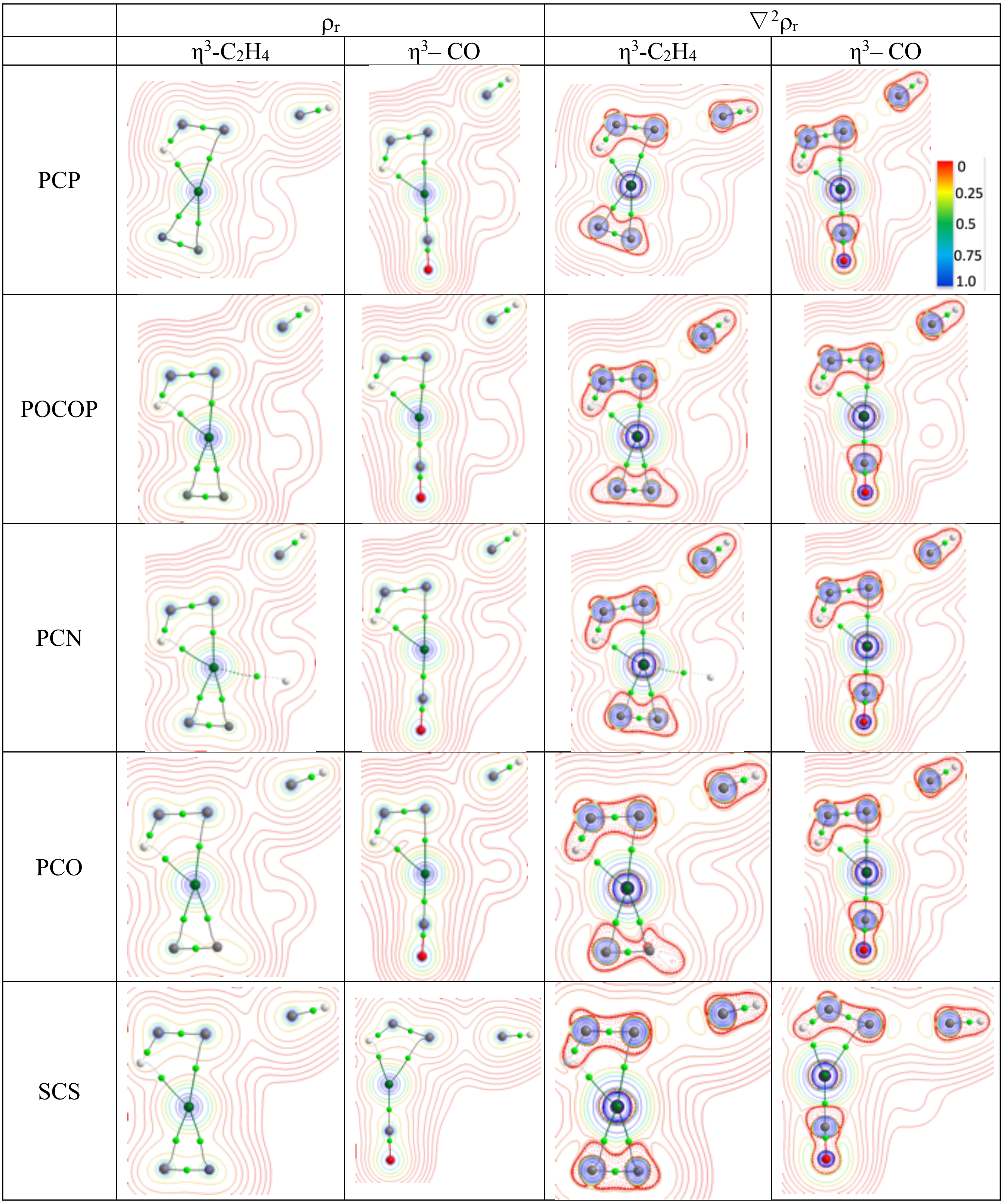

Figure S3. Density (ρ_r) and Laplacian of electron density ($\nabla^2\rho_r$) in η^3 agostic Rh(I) complexes with ethylene and carbonyl ancillary ligands. Charge accumulation - dotted lines, charge depletion - solid lines.

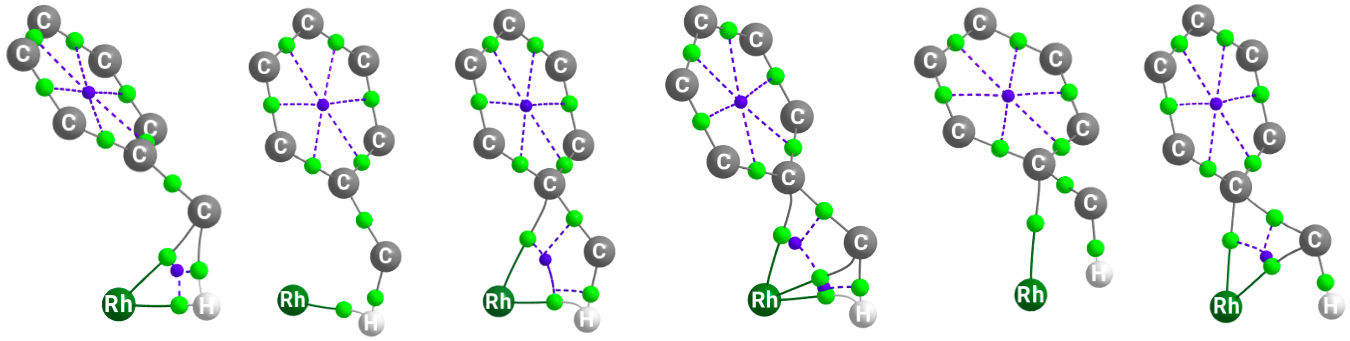

TS$_{C\text{-}H}$ ΔE=3.07	ν=178; f_c=0.054 ΔE=3.36	η^3 agostic Rh-POCOP ΔE=0	ν=313;f_c=0.094 ΔE=12.04	ν=278; f_c=0.122 ΔE=2.27	TS$_{C\text{-}C}$ ΔE=4.90

Figure S4. Molecular graphs of η^3-agostic Rh-POCOP complex in the equilibrium geometry (ΔE=0), in selected nonequilibrium geometries along low frequency vibrational mode, and in TS for C-C and C-H bond cleavage TS$_{C\text{-}C}$ and TS$_{C\text{-}H}$. Vibrational frequencies ν in cm^{-1}, force constant f_c in mDyne/Å, relative energies ΔE with respect to the η^3 agostic complex at the PBE0/TZVPP level of theory in kcal/mol.
